# Association of radiation exposure and blood lipid derived indices with hypertension risk in radiation workers

**DOI:** 10.3389/fpubh.2026.1870426

**Published:** 2026-06-26

**Authors:** Ruihua Ma, Yi Wang, Jiali Chen, Jingyan Gao, Lina Yan, Xia Gao

**Affiliations:** Department of Epidemiology and Health Statistics & Hebei Province Key Laboratory of Environment and Human Health, School of Public Health, Hebei Medical University, Shijiazhuang, Hebei, China

**Keywords:** blood lipids and derived indices, cohort study, cross-sectional study, hypertension, radiation exposure, radiation workers

## Abstract

**Background:**

This study aimed to explore the relationships between occupational radiation exposure, serum lipid levels and hypertension, and to assess the potential intermediate role of lipid parameters in the relevant association.

**Methods:**

A cross-sectional study (2,548 subjects) and a retrospective cohort study (484 subjects) were conducted among radiation workers who underwent physical examinations at a medical center in Shijiazhuang from October 2021 to March 2025. In the cross-sectional study, multivariate Logistic regression, restricted cubic spline (RCS) and mediation analysis were used to explore the association between radiation exposure and hypertension, as well as the mediating role of lipid parameters. In the cohort study, Cox proportional hazards regression, Kaplan–Meier curve, receiver operating characteristic (ROC) curve, stratified analysis and sensitivity analysis were applied to evaluate the association and predictive performance of baseline lipid indices for incident hypertension.

**Results:**

The cross-sectional analysis showed that, after adjustment for confounders, radiation working years, high working-year group and industrial radiation exposure were risk factors for hypertension (all *p* < 0.05); high-density lipoprotein cholesterol (HDL-C) was a protective factor, while elevated total cholesterol (TC), triglyceride (TG), low-density lipoprotein cholesterol (LDL-C), fasting blood glucose/HDL-C (FBG/HDL-C), TC/HDL-C and non-HDL-C/HDL-C ratio (NHHR) were risk factors. Mediation analysis indicated that TC, TG and LDL-C might mediate the positive association between long working years and hypertension, whereas HDL-C mediated the inverse association of working years and industrial radiation with hypertension. The cohort analysis revealed that elevated baseline TG, FBG/HDL-C, TC/HDL-C and NHHR increased the risk of incident hypertension, while elevated HDL-C decreased such risk; FBG/HDL-C and TC/HDL-C showed the best predictive performance; these associations were more prominent in males and overweight individuals.

**Conclusion:**

Prolonged radiation working years and industrial radiation exposure are associated with an increased risk of hypertension in radiation workers, and lipid profiles may play a mediating role. FBG/HDL-C, TC/HDL-C and NHHR are superior to conventional lipid indices in predicting hypertension. Male and overweight radiation workers are key targets for hypertension prevention and control.

## 1 Introduction

Hypertension is a highly prevalent cardiometabolic disease globally and a major risk factor for severe complications, including stroke, coronary heart disease, and renal failure (1). The latest national epidemiological survey indicates that the prevalence of hypertension among Chinese adults continues to rise, while the rates of awareness, treatment, and control remain relatively low, posing a severe challenge to hypertension prevention and control. Thus, there is an urgent need to conduct precise risk assessment and early intervention for key occupational populations (2, 3).

Ionizing radiation is widely applied in medical diagnosis and treatment, the nuclear industry, and scientific research (4, 5). With the popularization of radiological technology, occupational radiation exposure among radiation workers has increased correspondingly (6). Although the overall occupational radiation dose in China shows a downward trend, medical-related radiation exposure remains at a relatively high level (7). Inadequate radiation protection measures further expose workers to long-term low-dose radiation, which significantly increases cardiovascular health hazards (8). Multiple cohort studies and systematic reviews have confirmed that occupational low-dose ionizing radiation exposure is associated with an elevated risk of non-neoplastic diseases, such as hypertension, coronary heart disease, and cataracts (9–14). Moreover, the risk of hypertension exhibits a significant dose–response relationship with prolonged radiation working years (15). The intensity of industrial radiation exposure is generally higher than that of medical radiation, resulting in more prominent cardiovascular health risks (16, 17).

Mechanistic studies suggest that ionizing radiation can induce oxidative stress, lipid peroxidation, vascular endothelial injury, and inflammatory responses, which disrupt lipid metabolism and lead to dyslipidemia (18, 19). Several epidemiological studies have verified that radiation workers commonly present lipid disorders, including elevated total cholesterol (TC), triglyceride (TG), low-density lipoprotein cholesterol (LDL-C), and decreased high-density lipoprotein cholesterol (HDL-C) (9, 20–23). Meanwhile, dyslipidemia is a well-established risk factor for the development and progression of hypertension (24–27). Both conventional lipid parameters and composite lipid ratios are closely associated with hypertension risk. Among these, composite indices such as fasting blood glucose/HDL-C (FBG/HDL-C), TC/HDL-C, and non-HDL-C/HDL-C ratio (NHHR) can more comprehensively reflect metabolic disorders and exhibit better predictive performance for cardiovascular diseases than single lipid indicators (28–32).

Currently, most studies focus on the association between radiation dose and hypertension, with few incorporating multi-dimensional occupational exposure characteristics such as radiation working years, job type, and physical examination type. In addition, studies systematically exploring the mediating effect and predictive value of conventional and composite lipid indices in radiation workers remain limited. Therefore, this study combined cross-sectional and retrospective cohort designs to investigate the association of occupational radiation exposure with hypertension, analyze the relationships of lipid profiles and their derived indices with incident hypertension as well as their potential mediating effects, and compare the predictive value of different lipid parameters for hypertension. The findings will provide a scientific basis for the early screening, risk assessment, and precise prevention and control of hypertension among radiation workers.

## 2 Methods

### 2.1 Study population

**Figure 1:**
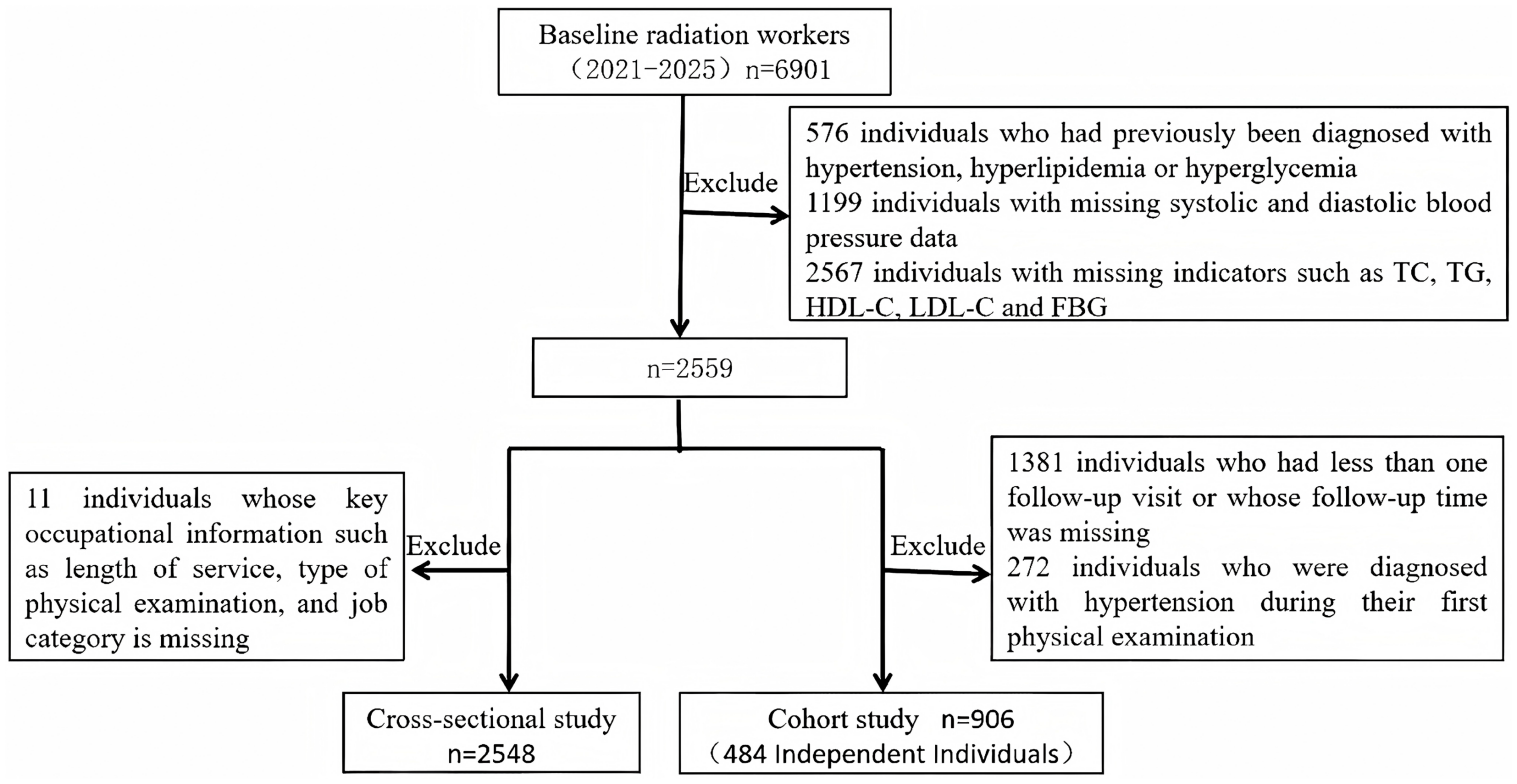
Flowchart of participant screening.

A combined cross-sectional and retrospective cohort design was adopted in this study. A total of 6,901 radiation workers who underwent occupational health examinations at a physical examination hospital in Shijiazhuang City, Hebei Province, between October 2021 and March 2025 were enrolled initially. For the cross-sectional study, 2,548 individuals were included after excluding those with a history of hypertension, hyperlipidemia and hyperglycemia, abnormal radiation exposure, and missing data on blood pressure, blood glucose, and lipid indicators. For the retrospective cohort study, the first physical examination was defined as the baseline, and incident hypertension as the endpoint; participants were followed up regularly until March 2025. After excluding those with pre-existing diseases, missing relevant indicators, and incomplete follow-up data, 484 eligible individuals were finally included. The participant screening flowchart is shown in Figure 1. This study was approved by the Ethics Committee of Hebei Medical University (approval number: HMU MEC 2024021), and all participants provided written informed consent.

### 2.2 Variables and definitions

This study is a retrospective analysis based on historical occupational medical examination records. No individual thermoluminescence dosimeter (TLD) dose data of the study population was collected. Therefore, occupational radiation exposure was characterized as a surrogate indicator of cumulative radiation burden through years of work, job category, and examination type. Participants’ self-reported radiation working years were used as a rough surrogate for cumulative exposure. Further verification of the self-reported work experience was conducted, and it was cross-checked with the official personnel records and occupational health examination files to reduce the recall bias. Working years were analyzed as both a continuous variable and generate a three-group variable based on the tertiles of the length of service (low: <2.09 years; middle: 2.09–8.99 years; high: >8.99 years). Job type (medical vs. industrial radiation) and examination type (pre-employment, during employment, post-employment) were also analyzed.

Systolic blood pressure (SBP) and diastolic blood pressure (DBP) were measured with an electronic sphygmomanometer after participants rested for 5–10 min. Two consecutive measurements were taken on the right upper arm at 1–2 min intervals, and the average value (accurate to 2 mmHg) was used. Hypertension was defined in accordance with the Chinese Guidelines for the Prevention and Treatment of Hypertension (2024 Revision), i.e., SBP ≥ 140 mmHg or DBP ≥ 90 mmHg.

Covariates included gender, age, education level, body mass index (BMI), smoking status, and drinking status; smoking and drinking were categorized as “yes” (past or current) or “no” (never), and overweight was defined as BMI ≥ 24 kg/m^2^. Serum TC, TG, LDL-C, HDL-C, and fasting blood glucose (FBG) were measured by an automatic biochemical analyzer. Lipid-derived indices (FBG/HDL-C, TC/HDL-C, NHHR) were calculated, with non-HDL-C = TC − HDL-C.

Exposure variables in the cross-sectional study included working years, working years groups, examination type, and job category; in the cohort study, they were baseline lipid profiles and their derived indices (continuous and tercile-grouped). Outcome variables were prevalent hypertension (cross-sectional study) and incident hypertension (cohort study). Covariates were treated as follows: age and BMI as continuous variables; gender, smoking, drinking, and job category as dichotomous variables; education level as a categorical variable. The cross-sectional model was adjusted for gender, age, education level, BMI, smoking, and drinking, while the cohort model was further adjusted for job category. Lipid profiles and their derived indices were regarded as potential mediators in the cross-sectional analysis to explore their mediating role between radiation exposure and hypertension.

### 2.3 Sample size justification

According to the criterion established by Peduzzi et al., multivariate regression models, including both logistic regression and Cox proportional hazards regression, require a minimum of 10 outcome events per covariate to ensure stable and reliable results (33, 34). In the present study, the cross-sectional model incorporated 7 covariates, thus necessitating a minimum of 70 outcome events; the cohort model comprised 8 covariates, requiring at least 80 events. Importantly, the actual sample sizes (2,548 participants in the cross-sectional study and 484 in the cohort study) substantially exceeded these minimum thresholds, confirming the adequacy of the study sample for the planned regression analyses.

### 2.4 Statistical analysis

For baseline characteristics, the Shapiro–Wilk test was used to test the normality of continuous variables: normally distributed data were expressed as mean ± standard deviation (*x* ± *s*), non-normally distributed data as median (interquartile range) [*M* (*QR*)], and categorical variables as frequency (percentage) [*n* (%)]. Between-group comparisons were conducted using Student’s t-test, Mann–Whitney U test, or *χ*^2^ test as appropriate.

In the cross-sectional analysis, multivariate Logistic regression was used to analyze the associations of radiation exposure and lipid indices with hypertension, reporting odds ratios (*OR*) and 95% confidence intervals (*CI*). Linear or ordinal Logistic regression was applied to explore the association between radiation exposure and lipid profiles, with three models constructed (Model 1 was unadjusted; Model 2 was adjusted for age and gender; Model 3 was further adjusted for age, gender, BMI, educational level, smoking status and drinking status) and multicollinearity tested via variance inflation factor (VIF). Restricted cubic spline (RCS) models were used to explore non-linear associations, with the optimal number of knots determined by the Akaike information criterion (AIC). Stratified analyses were performed by gender, overweight status, smoking, drinking, working years, and job category to assess interaction effects, and mediation analysis was used to explore the correlation, with 95% CIs estimated by the Bootstrap method (1,000 resamplings). Given the limitation of the cross-sectional design in this study, the causal temporal relationship could not be established.

In the cohort analysis, Cox proportional hazards regression was used to analyze the associations of baseline lipid indices with incident hypertension, reporting hazard ratios (*HR*) and 95% *CI*, with three models constructed, Model 1 was unadjusted; Model 2 was adjusted for age and gender; Model 3 was further adjusted for age, gender, BMI, educational level, smoking status, drinking status and occupational category. The proportional hazards assumption was tested using Schoenfeld residuals, and multicollinearity was evaluated via VIF. Kaplan–Meier curves were plotted to describe the cumulative incidence of hypertension, with between-group differences tested by the Log-rank test. Predictive performance was assessed using receiver operating characteristic (ROC) curves. RCS models were used to explore non-linear associations, further verified by segmented Cox regression. Stratified analyses were performed by gender, BMI, working years, and job category, with interaction effects tested using the likelihood ratio test. Sensitivity analysis was conducted by excluding participants with follow-up duration<6 months to verify the robustness of the results.

## 3 Results

### 3.1 Results of the cross-sectional study

#### 3.1.1 Baseline characteristics of study participants

The cross-sectional study included 2,548 radiation workers, among whom 567 were diagnosed with hypertension (see Table 1). The median age (interquartile range) of the study population was 35.00(11.00) years, with 59.81% male and 40.19% female participants. Between-group comparisons revealed that participants in the hypertension group were significantly older, and had higher proportions of male sex, lower educational attainment, smoking, alcohol consumption, and overweight status compared with the normotensive group (all *p* < 0.001). Radiation-related indicators, including working years, working-year groups, physical examination type, and job category, were also significantly associated with the prevalence of hypertension (all *p* < 0.001). With regard to lipid parameters, the hypertension group exhibited significantly higher levels and higher abnormal rates of TC, TG, LDL-C, FBG/HDL-C, TC/HDL-C, and NHHR, as well as a significantly lower level of HDL-C than the normotensive group (all *p* < 0.001).

**Table 1:** Basic characteristics of cross-sectional radiation workers.

Variable	Total population (*n* = 2,548)	Hypertension		*Z*/*x^2^*	*p*
		No(*n* = 1981)	Yes(*n* = 567)		
Age, years, *M*(*QR*)	35.00(11.00)	34.00(11.00)	39.00(14.00)	135.42	**<0.001**
Female, *n*(%)	1,024(40.19)	948(47.80)	76(13.40)	217.66	**<0.001**
Educational level, *n* (%)				7.38	**0.027**
Junior high school or below	1,065(41.80)	806(40.69)	259(45.68)		
Senior high school / polytechnic	1,018(39.95)	794(40.08)	224(39.51)		
Junior college or above	465(18.25)	381(19.23)	84(14.81)		
Smoking, *n*(%)	347(13.62)	223(11.26)	124(21.87)	42.20	**<0.001**
Alcohol drinking, *n*(%)	772(30.30)	529(26.70)	243(42.86)	54.47	**<0.001**
Overweight, *n*(%)				56.22	**<0.001**
No	1,045(41.01)	893(45.08)	152(26.81)		
Yes	1,425(55.93)	1,038(52.40)	387(68.25)		
NA	78(3.06)	50(2.52)	28(4.94)		
Working years, years, *M*(*QR*)	4.64(9.65)	4.00(9.00)	7.02(13.41)	134.15	**<0.001**
Job classification, *n*(%)				48.22	**<0.001**
Medical	1,606(63.03)	1,319(66.58)	287(50.62)		
Industrial	942(36.97)	662(33.42)	280(49.38)		
Working years groups, *n*(%)				49.19	**<0.001**
Low (<2.09)	859(33.71)	713(35.99)	146(25.75)		
Middle (2.09–8.99)	849(33.32)	683(34.48)	166(29.280)		
High (>8.99)	840(32.97)	585(29.53)	255(45.97)		
Type of physical examination, *n*(%)				23.41	**<0.001**
Pre-employment	606(23.78)	508(25.64)	98(17.28)		
During employment	1806(70.88)	1,358(68.55)	448(79.01)		
Post-employment	136(5.34)	115(5.81)	21(3.71)		
TC(mmol/L)	4.57(1.14)	4.50(1.15)	4.77(1.16)	134.34	**<0.001**
TG(mmol/L)	1.14(1.00)	1.02(0.89)	1.58(1.20)	139.14	**<0.001**
HDL-C(mmol/L)	1.28(0.42)	1.32(0.44)	1.20(0.32)	117.74	**<0.001**
LDL-C(mmol/L)	2.87(1.01)	2.80(0.98)	3.14(0.98)	136.30	**<0.001**
FBG/HDL-C	4.00(1.53)	3.83(1.49)	4.51(1.45)	138.96	**<0.001**
TC/HDL-C	3.54(1.40)	3.39(1.29)	4.03(1.32)	139.86	**<0.001**
NHHR	2.54(1.40)	2.39(1.29)	3.03(1.32)	139.87	**<0.001**
TC groups, *n*(%)				48.34	**<0.001**
Low (<4.21)	856(33.59)	724(36.55)	132(23.28)		
Middle (4.21–4.95)	846(33.20)	660(33.32)	186(32.80)		
High (>4.95)	846(33.21)	597(30.13)	249(43.92)		
TG groups, *n*(%)				139.49	**<0.001**
Low (<0.87)	859(33.71)	764(38.57)	95(16.75)		
Middle (0.87–1.52)	840(32.97)	663(33.47)	177(31.22)		
High (>1.52)	849(33.32)	554(27.96)	295(52.03)		
HDL-C groups, *n*(%)				79.73	**<0.001**
Low (<1.16)	857(33.63)	604(30.49)	253(44.62)		
Middle (1.16–1.43)	871(34.18)	655(33.06)	216(38.10)		
High (>1.43)	820(32.19)	722(36.45)	98(17.28)		
LDL-C groups, *n*(%)				79.71	**<0.001**
Low (<2.57)	855(33.56)	741(37.41)	114(20.11)		
Middle (2.57–3.21)	855(33.56)	665(33.57)	190(33.51)		
High (>3.21)	838(32.88)	575(29.02)	263(46.38)		
FBG/HDL-C groups, *n*(%)				116.73	**<0.001**
Low (<3.53)	858(33.67)	762(38.47)	96(16.93)		
Middle (3.53–4.52)	846(33.20)	653(32.96)	193(34.04)		
High (>4.52)	844(33.13)	566(28.57)	278(49.03)		
TC/HDL-C groups, *n*(%)				141.41	**<0.001**
Low (<3.12)	851(33.40)	757(38.21)	94(16.58)		
Middle (3.12–3.99)	853(33.48)	675(34.07)	178(31.39)		
High (>3.99)	844(33.12)	549(27.72)	295(52.03)		
NHHR groups, *n*(%)				142.22	**<0.001**
Low (<2.12)	851(33.40)	757(38.21)	94(16.58)		
Middle (2.12–2.99)	853(33.48)	675(34.07)	178(31.39)		
High (>2.99)	844(33.12)	549(27.72)	295(52.03)		

BMI, body mass index; TC, total cholesterol; TG, triglyceride; HDL-C, high-density lipoprotein cholesterol; LDL-C, low-density lipoprotein cholesterol; FBG/HDL-C, fasting blood glucose to high-density lipoprotein cholesterol ratio; TC/HDL-C, total cholesterol to high-density lipoprotein cholesterol ratio; NHHR, non-high-density lipoprotein cholesterol to high-density lipoprotein cholesterol ratio. Bold values indicate statistically significant results with a two-tailed *p*-value < 0.05.

#### 3.1.2 Association between radiation exposure and hypertension risk

Multivariate logistic regression results were summarized in Table 2. After adjusting for gender, age, BMI, education, smoking, and alcohol consumption, working years (*OR* = 1.014, 95%*CI*: 1.001–1.027), the high working-year group vs. low level (*OR* = 1.385, 95%*CI*: 1.058–1.816), and industrial radiation vs. medical radiation (*OR* = 2.003, 95%*CI*: 1.596–2.516) were identified as independent risk factors for hypertension (all *p* < 0.05). No significant association was found between examination type and hypertension risk (*p* > 0.05). Restricted cubic spline (RCS) analysis indicated no nonlinear relationship between working years and hypertension risk (Supplementary Figure 1A).

**Table 2:** Analysis of the association between radiation exposure and hypertension.

Radiation exposure	Hypertension *OR*(95% *CI*)		
	Model 1	Model 2	Model 3
Working years	**1.039(1.029,1.050)**	1.012(0.999,1.024)	**1.014(1.001,1.027)**
Working years groups			
Low (<2.09)	Reference	Reference	Reference
Middle (2.09–8.99)	1.187(0.928,1.519)	1.068(0.825,1.382)	1.004(0.770,1.308)
High (>8.99)	**2.129(1.692,2.686)**	**1.397(1.075,1.819)**	**1.385(1.058,1.816)**
Type of physical examination			
Pre-employment	Reference	Reference	Reference
During employment	**1.710(1.349,2.186)**	1.209(0.936,1.570)	1.137(0.875,1.487)
Post-employment	0.947(0.554,1.554)	0.703(0.399,1.186)	0.645(0.351,1.132)
Job classification			
Medical	Reference	Reference	Reference
Industrial	**1.944(1.609,2.349)**	**1.903(1.533,2.366)**	**2.003(1.596,2.516)**

Model 1: Unadjusted; Model 2: Adjusted for sex and age; Model 3: Further adjusted for BMI, educational level, smoking and alcohol drinking on the basis of Model 2. Bold values indicate statistically significant results with a two-tailed *p*-value < 0.05.

#### 3.1.3 Association between radiation exposure and lipid profiles

Associations of radiation exposure with lipid indices are presented in Table 3. Working years, working-year groups, and examination type were positively associated with TC level and grade, with longer employment duration linked to higher TC. The high working-year group showed elevated TG levels, while the middle group had higher TG grade. Working years and job type were positively correlated with HDL-C level and grade. Working-year groups and examination type were positively associated with LDL-C level and grade.

**Table 3:** Association analysis of radiation exposure with continuous and three-group lipid levels and derived variables [*β*/*OR*(95% *CI*)].

Subgroup variables	Working years	Working years groups		*P* for trend	Type of physical examination		*P* for trend	Job classification
		Middle^a^	High^a^		During employment^b^	Post-employment^b^		Industrial^c^
TC	**0.007** **(0.002,0.011)**	**0.089** **(0.012,0.167)**	**0.138** **(0.048,0.227)**	**0.002**	**0.135** **(0.057,0.214)**	0.117 (−0.053,0.288)	**0.004**	0.064 (−0.010,0.137)
TG	9.44e^−05^ (−0.006,0.006)	0.066 (−0.014,0.146)	**0.167** **(0.058,0.277)**	**0.002**	0.057 (−0.024,0.138)	0.049 (−0.212,0.311)	0.309	0.028 (−0.059,0.115)
HDL-C	**0.003** **(0.001,0.004)**	−0.013 (−0.041,0.014)	0.004 (−0.026,0.035)	0.857	0.020 (−0.008,0.047)	0.012 (−0.038,0.062)	0.277	**0.027** **(0.002,0.052)**
LDL-C	0.004 (−0.001,0.008)	**0.083** **(0.015,0.150)**	**0.097** **(0.019,0.175)**	**0.010**	**0.115** **(0.46,0.183)**	0.100 (−0.042,0.241)	**0.005**	0.022 (−0.042,0.089)
FBG/HDL-C	−0.008 (−0.015,-0.0002)	−0.072 (−0.234,0.089)	−0.050 (−0.209,0.108)	0.511	−0.148 (−0.316,0.019)	−0.112 (0.462,0.237)	0.107	−0.086 (−0.237,0.065)
TC/HDL-C	−0.002 (−0.007,0.004)	0.081 (−0.023,0.186)	0.094 (−0.012,0.201)	0.078	0.029 (−0.088,0.145)	0.056 (−0.151,0.262)	0.497	−0.037 (−0.139,0.064)
NHHR	−0.002 (−0.007,0.004)	0.081 (−0.023,0.186)	0.094 (−0.012,0.201)	0.078	0.029 (−0.088,0.145)	0.056 (−0.151,0.262)	0.497	−0.037 (−0.139,0.064)
TC groups	**1.013** **(1.003,1.024)**	**1.259** **(1.054,1.500)**	**1.407** **(1.155,1.710)**	**0.001**	**1.363** **(1.142,1.629)**	1.392 (0.977,1.982)	**0.002**	1.070 (0.909,1.260)
TG groups	0.995 (0.984,1.005)	**1.262** **(1.054,1.510)**	1.187 (0.972,1.450)	0.066	1.118 (0.934,1.338)	0.902 (0.628,1.295)	0.689	1.057 (0.895,1.250)
HDL-C groups	**1.019** **(1.008,1.029)**	0.850 (0.709,1.020)	1.011 (0.827,1.240)	0.949	1.060 (0.885,1.269)	1.178 (0.827,1.679)	0.355	1.150 (0.974,1.360)
LDL-C groups	**1.013** **(1.008,1.023)**	**1.308** **(1.094,1.560)**	**1.363** **(1.118,1.660)**	**0.002**	**1.401** **(1.171,1.675)**	**1.584** **(1.108,2.263)**	**0.0002**	1.080 (0.918,1.270)
FBG/HDL-C groups	**0.985** **(0.974,0.996)**	1.015 (0.846,1.220)	0.896 (0.732,1.100)	0.318	**0.812** **(0.676,0.976)**	0.751 (0.527,1.070)	**0.024**	0.849 (0.718,1.000)
TC/HDL-C groups	0.993 (0.982,1.003)	**1.310** **(1.091,1.570)**	1.197 (0.978,1.460)	0.057	1.183 (0.984,1.421)	0.963 (0.674,1.377)	0.365	0.932 (0.788,1.100)
NHHR groups	0.993 (0.982,1.003)	**1.310** **(1.091,1.570)**	1.197 (0.978,1.460)	0.057	1.183 (0.984,1.421)	0.963 (0.674,1.377)	0.365	0.932 (0.788,1.100)

a For working years groups, the medium and high level groups were both referenced to the low level group.

b For type of physical examination, during employment and post-employment were both referenced to pre-employment.

c For job classification, industrial radiation workers were referenced to medical radiation workers. Bold values indicate statistically significant results with a two-tailed *p*-value < 0.05.

FBG/HDL-C grade increased with longer working years and was higher in workers undergoing periodic examinations than in pre-employment examinees. The middle working-year group had higher TC/HDL-C and NHHR grades than the low group. RCS analysis revealed significant nonlinear associations between working years and most lipid and derived indices, except for FBG/HDL-C (Supplementary Figures 1B–H).

#### 3.1.4 Association between lipid profiles and hypertension risk

Multivariate logistic regression results are displayed in Supplementary Table 1. After covariate adjustment, higher HDL-C level and grade were inversely associated with hypertension risk (*OR* = 0.538, 95%*CI*: 0.366–0.787; *OR* = 0.622, 95%*CI*: 0.464–0.830), suggesting a protective effect (both *p* < 0.05). Elevated levels and grades of TC, TG, LDL-C, FBG/HDL-C, TC/HDL-C, and NHHR were positively associated with hypertension prevalence (all *p* < 0.05), with the high TG group showing the strongest effect (*OR* = 2.230, 95%*CI*: 1.693–2.955). RCS analysis confirmed nonlinear relationships for TG, FBG/HDL-C, TC/HDL-C, and NHHR (Supplementary Figures 1B–H).

#### 3.1.5 Subgroup analysis

Subgroup analysis results are shown in Supplementary Table 2. The associations between radiation-related indicators and hypertension varied across gender, age, education, smoking, drinking, and overweight status. After adjustment, working years were positively associated with hypertension among males, individuals aged ≥35 years, non-smokers, and drinkers (all *p* < 0.05). The high working-year group was associated with higher hypertension risk among males, those aged ≥35 years, participants with senior high school/technical secondary education, non-smokers, drinkers, and overweight individuals (all *p* < 0.05). For examination type, only overweight workers undergoing periodic examinations had higher hypertension risk than pre-employment workers (*OR* = 1.663, 95%*CI*: 1.201–2.332, *p* < 0.05). Industrial radiation was consistently associated with higher hypertension risk than medical radiation across all subgroups (all *p* < 0.05).

#### 3.1.6 Mediation effects of lipid profiles

**Figure 2:**
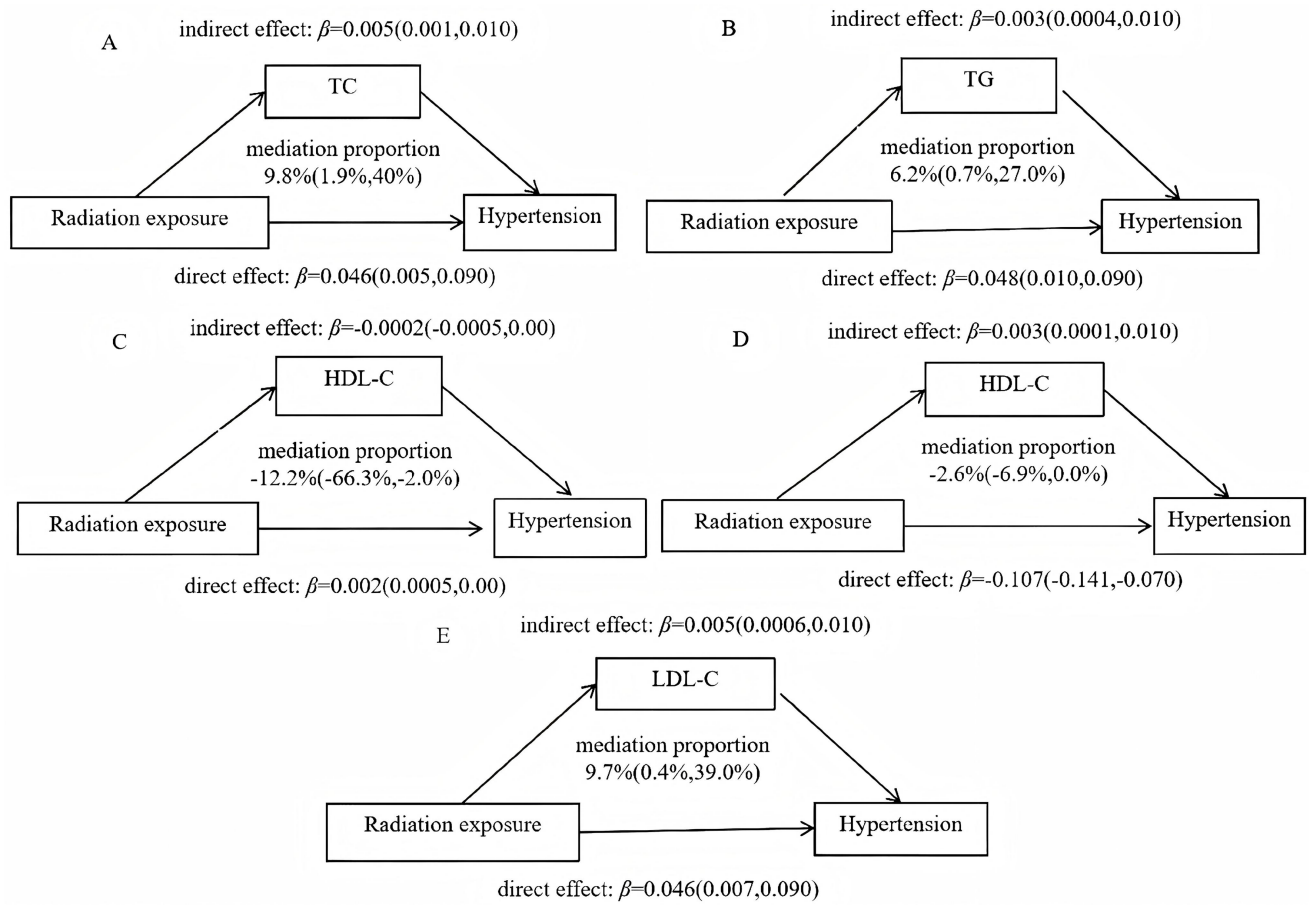
Mediating Effects of Lipid Indices on Radiation-associated Hypertension. The moderating effects of TC **(A)** and TG **(B)** on the association between radiation working years groups and hypertension; the moderating effect of HDL-C **(C)** on the association between radiation working years and hypertension; the moderating effect of HDL-C **(D)** on the association between job category and hypertension; the moderating effect of LDL-C **(E)** on the association between radiation working years groups and hypertension (*p* < 0.05).

Mediation analysis results are illustrated in Figure 2. After adjustment, HDL-C mediated a partial negative effect between working years and hypertension, accounting for 12.2% of the total association (total effect *β* = 0.002, 95%*CI*: 0.0003–0.004; direct effect *β* = 0.002, 95%*CI*: 0.0005–0.004; indirect effect *β* = −0.0002, 95%*CI*: −0.0005 - 0.0000; all *p* < 0.05). TC, TG, and LDL-C partially mediated positive associations between the high working-year group and hypertension, with mediation proportions of 9.8, 6.2, and 9.7%, respectively (all *p* < 0.05). HDL-C also exerted a small negative mediating effect (2.6%) between industrial radiation and hypertension (total effect *β* = −0.104, 95%*CI*: −0.138–−0.070; direct effect *β* = −0.107, 95%*CI*: −0.141–−0.070; indirect effect *β* = 0.003, 95%*CI*: 0.0001–0.010; *p* < 0.05). No significant mediation was observed for other lipid-derived indices.

### 3.2 Results of the cohort study

#### 3.2.1 Baseline characteristics of cohort participants

The cohort included 484 radiation workers with a median follow-up of 1.39 years. During follow-up, 118 participants developed incident hypertension (Table 4). The mean age was 34.00 (11.00) years, with 56.4% males and 43.6% females. Participants who developed hypertension were significantly older, more likely to be male, and had a higher drinking rate than those who remained normotensive (all *p* < 0.05).

**Table 4:** Comparison of the basic characteristics of radiation workers with or without hypertension.

Variable	Total population (*n* = 484)	Hypertension		*Z*/*x*^2^	*p*
		否(*n* = 366)	是(*n* = 118)		
Age, years, *M*(*QR*)	34.00(11.00)	33.00(10.00)	35.00(15.00)	1.99	**0.047**
Female, *n*(%)	211(43.60)	184(50.27)	27(22.88)	26.12	**<0.001**
Educational level, *n* (%)				0.92	0.632
Junior high school or below	210(43.39)	161(43.99)	49(41.53)		
Senior high school / polytechnic	221(45.66)	163(44.54)	58(49.15)		
Junior college or above	53(10.95)	42(11.48)	11(9.32)		
Smoking, *n*(%)	62(12.81)	45(12.30)	17(14.41)	0.19	0.661
Alcohol drinking, *n*(%)	140(28.93)	92(25.14)	48(40.68)	9.74	**0.002**
BMI groups, *n*(%)				3.15	0.076
No	200(41.32)	160(43.72)	40(33.90)		
Yes	284(58.68)	206(56.28)	78(66.10)		
Follow-up time, years, *M*(*QR*)	1.39(0.96)	1.44(0.97)	1.31(0.83)	0.99	0.320
TC(mmol/L)	4.53(1.11)	4.49(1.11)	4.64(1.10)	1.32	0.186
TG(mmol/L)	1.01(0.95)	0.97(0.80)	1.42(1.44)	4.31	**<0.001**
HDL-C(mmol/L)	1.31(0.45)	1.37(0.45)	1.21(0.40)	4.44	**<0.001**
LDL-C(mmol/L)	2.84(0.94)	2.79(0.97)	2.99(0.91)	1.94	0.052
FBG/HDL-C	3.84(1.48)	3.71(1.33)	4.49(1.48)	5.24	**<0.001**
TC/HDL-C	3.41(1.24)	3.33(1.07)	3.84(1.35)	4.96	**<0.001**
NHHR	2.41(1.24)	2.33(1.07)	2.84(1.35)	4.96	**<0.001**
TC groups, *n*(%)				1.29	0.525
Low (<4.20)	161(33.26)	126(34.43)	35(29.66)		
Middle (4.20–4.91)	161(33.26)	122(33.33)	39(33.05)		
High (>4.91)	162(33.47)	118(32.24)	44(37.29)		
TG groups, *n*(%)				23.70	**<0.001**
Low (<0.84)	160(33.06)	131(35.79)	29(24.58)		
Middle (0.84–1.39)	159(32.85)	132(36.07)	27(22.88)		
High (>1.39)	165(34.09)	103(28.14)	62(52.54)		
HDL-C groups, *n*(%)				21.90	**<0.001**
Low (<1.18)	160(33.06)	102(27.87)	58(49.15)		
Middle (1.18–1.48)	160(33.06)	123(33.61)	37(31.36)		
High (>1.48)	164(33.88)	141(38.52)	23(19.49)		
LDL-C groups, *n*(%)				4.72	0.095
Low (<2.54)	161(33.26)	130(35.52)	31(26.27)		
Middle (2.54–3.14)	161(33.26)	122(33.33)	39(33.05)		
High (>3.14)	162(33.47)	114(31.15)	48(40.68)		
FBG/HDL-C groups, *n*(%)				32.89	**<0.001**
Low (<3.43)	161(33.26)	136(37.16)	25(21.19)		
Middle (3.43–4.36)	161(33.26)	133(36.34)	28(23.73)		
High (>4.36)	162(33.47)	97(26.50)	65(55.08)		
TC/HDL-C groups, *n*(%)				36.43	**<0.001**
Low (<3.02)	161(33.26)	139(37.98)	22(18.64)		
Middle (3.02–3.76)	161(33.26)	131(35.79)	30(25.42)		
High (>3.76)	162(33.47)	96(26.23)	66(55.93)		
NHHR groups, *n*(%)				36.43	**<0.001**
Low (<2.02)	161(33.26)	139(37.98)	22(18.64)		
Middle (2.02–2.76)	161(33.26)	131(35.79)	30(25.42)		
High (>2.76)	162(33.47)	96(26.23)	66(55.93)		

BMI, body mass index; TC, total cholesterol; TG, triglyceride; HDL-C, high-density lipoprotein cholesterol; LDL-C, low-density lipoprotein cholesterol; FBG/HDL-C, fasting blood glucose to high-density lipoprotein cholesterol ratio; TC/HDL-C, total cholesterol to high-density lipoprotein cholesterol ratio; NHHR, non-high-density lipoprotein cholesterol to high-density lipoprotein cholesterol ratio. Bold values indicate statistically significant results with a two-tailed *p*-value < 0.05.

No significant between-group differences were observed in education, smoking, BMI category, or follow-up duration (all *p* > 0.05). Levels and grades of TG, HDL-C, FBG/HDL-C, TC/HDL-C, and NHHR were significantly associated with incident hypertension (all *p* < 0.05).

#### 3.2.2 Association between lipid profiles and incident hypertension

Cox proportional hazards regression results (validated using Schoenfeld residuals) are shown in Table 5. After adjustment, each 1-unit increase in TG, FBG/HDL-C, TC/HDL-C, and NHHR was associated with 19.5, 7.7, 9.9, and 10.0% higher hypertension risk, respectively (*HR* = 1.195, 1.077, 1.099, 1.100; all *p* < 0.05). Each 1-unit increase in HDL-C was associated with a 66.2% lower risk (*HR* = 0.338, *p* < 0.05).

**Table 5:** Association analysis of continuous or the three-classification indicators of blood lipids or their derived variables with the risk of hypertension.

Blood lipids and their derivative indicators (mmol/L)	Number of participants	Cases / person-years	*HR*(95% *CI*)		
			Model 1	Model 2	Model 3
TC	484	118/682	1.050(0.843,1.308)	0.888(0.696,1.133)	0.903(0.706,1.156)
TG	484	118/682	**1.354(1.184,1.549)**	**1.198(1.030,1.394)**	**1.195(1.023,1.396)**
HDL-C	484	118/682	**0.264(0.147,0.473)**	**0.360(0.187,0.693)**	**0.338(0.172,0.664)**
LDL-C	484	118/682	1.172(0.900,1.527)	0.903(0.676,1.206)	0.932(0.695,1.250)
FBG/HDL-C	484	118/682	**1.075(1.038,1.114)**	**1.065(1.020,1.112)**	**1.077(1.029,1.127)**
TC/HDL-C	484	118/682	**1.096(1.043,1.152)**	**1.082(1.018,1.150)**	**1.099(1.031,1.172)**
NHHR	484	118/682	**1.096(1.043,1.152)**	**1.082(1.018,1.150)**	**1.100(1.031,1.173)**
TC groups					
Low (<4.20)	161	35/221	Reference	Reference	Reference
Middle (4.20–4.91)	161	39/228	1.114(0.705,1.759)	0.943(0.595,1.495)	0.921(0.577,1.470)
High (>4.91)	162	44/232	1.106(0.708,1.729)	0.768(0.477,1.237)	0.779(0.483,1.258)
*P* for trend			0.919	0.986	0.931
TG groups					
Low (<0.84)	160	29/226	Reference	Reference	Reference
Middle (0.84–1.39)	159	27/226	0.938(0.555,1.585)	0.768(0.452,1.307)	0.762(0.447,1.297)
High (>1.39)	165	62/230	**2.131(1.369,3.316)**	1.377(0.854,2.220)	1.367(0.845,2.210)
*P* for trend			0.904	0.801	0.725
HDL-C groups					
Low (<1.18)	160	58/216	Reference	Reference	Reference
Middle (1.18–1.48)	160	37/218	**0.601(0.398,0.908)**	0.673(0.443,1.023)	**0.639(0.418,0.977)**
High (>1.48)	164	23/248	**0.293(0.179,0.480)**	**0.372(0.221,0.626)**	**0.362(0.214,0.613)**
*P* for trend			0.526	0.539	0.479
LDL-C groups					
Low (<2.54)	161	31/220	Reference	Reference	Reference
Middle (2.54–3.14)	161	39/227	1.215(0.758,1.949)	0.857(0.527,1.395)	0.879(0.539,1.433)
High (>3.14)	162	48/236	1.293(0.821,2.037)	0.831(0.512,1.349)	0.866(0.532,1.409)
*P* for trend			0.754	0.831	0.905
FBG/HDL-C groups					
Low (<3.43)	161	25/237	Reference	Reference	Reference
Middle (3.43–4.36)	161	28/224	1.235(0.716,2.132)	1.019(0.582,1.783)	0.996(0.565,1.754)
High (>4.36)	162	65/221	**3.207(2.002,5.136)**	**2.181(1.296,3.670)**	**2.205(1.292,3.763)**
*P* for trend			0.412	0.482	0.456
TC/HDL-C groups					
Low (<3.02)	162	22/229	Reference	Reference	Reference
Middle (3.02–3.76)	161	30/232	1.311(0.756,2.275)	1.306(0.589,1.822)	1.039(0.589,1.831)
High (>3.76)	161	66/221	**3.191(1.967,5.176)**	**2.128(1.259,3.598)**	**2.189(1.293,3.704)**
*P* for trend			0.354	0.326	0.281
NHHR groups					
Low (<2.02)	161	22/229	Reference	Reference	Reference
Middle (2.02–2.76)	161	30/231	1.312(0.756,2.276)	1.037(0.590,1.822)	1.035(0.587,1.824)
High (>2.76)	162	66/222	**3.189(1.966,5.173)**	**2.127(1.258,3.596)**	**2.180(1.289,3.689)**
*P* for trend			0.351	0.325	0.275

Model 1: Unadjusted; Model 2: Adjusted for sex and age; Model 3: Further adjusted for educational level, BMI, smoking, alcohol drinking and job category on the basis of Model 2. Bold values indicate statistically significant results with a two-tailed *p*-value < 0.05.

Compared with the low HDL-C group, the middle and high groups showed 36.1 and 63.8% lower risk (*HR* = 0.639, 0.362; both *p* < 0.05). The high groups of FBG/HDL-C, TC/HDL-C, and NHHR were associated with 120.5, 118.9, and 118.0% higher risk than the corresponding low groups (*HR* = 2.205, 2.189, 2.180; all *p* < 0.05). No significant linear trends were detected (all *P* for trend > 0.05).

**Figure 3:**
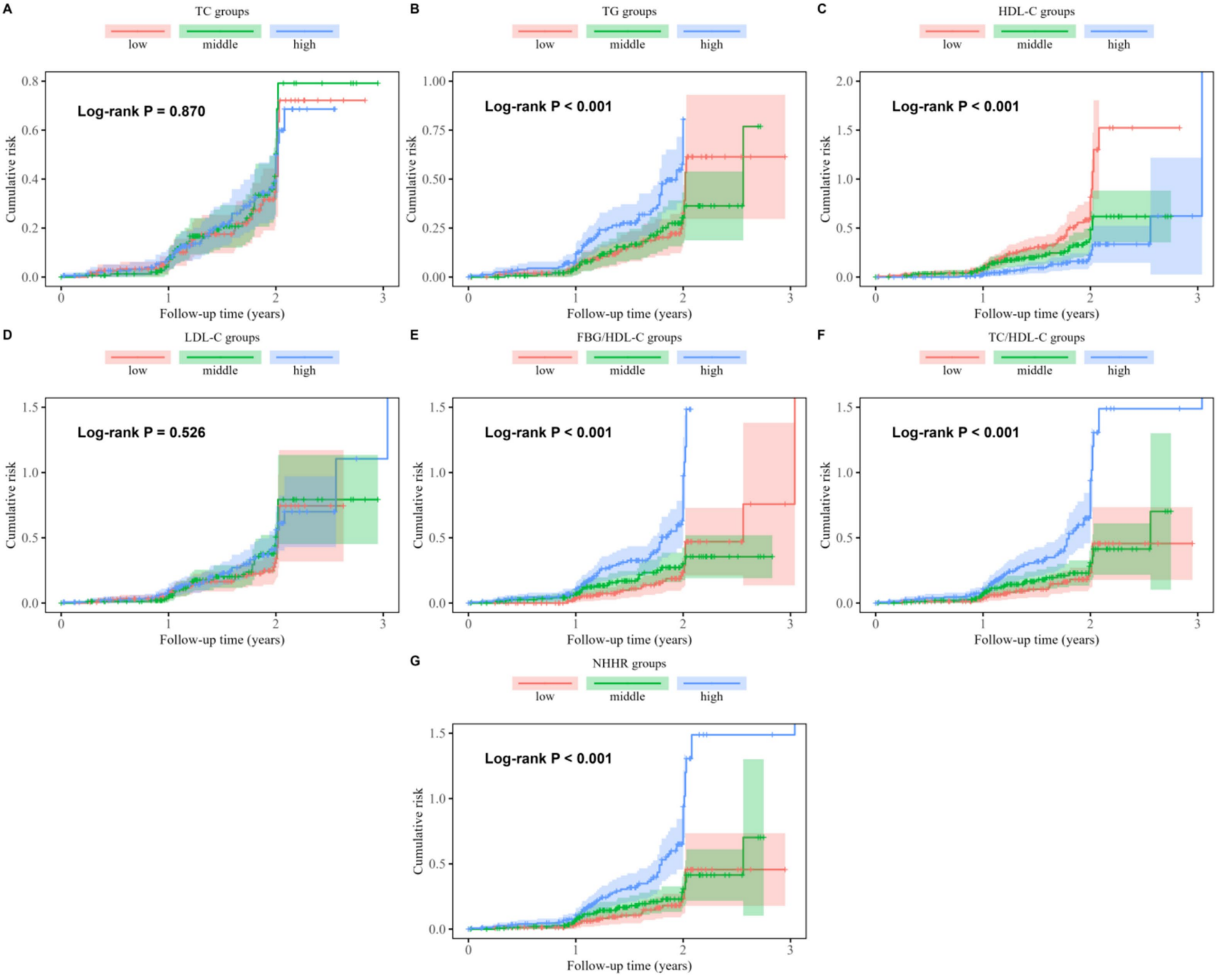
Cumulative hypertension hazard by lipid tertile groups. Cumulative risk plots of radiation workers by TC groups **(A)**, TG groups **(B)**, HDL-C groups **(C)**, LDL-C groups **(D)**, FBG/HDL-C groups **(E)**, TC/HDL-C groups **(F)**, and NHHR groups **(G)**.

**Figure 4:**
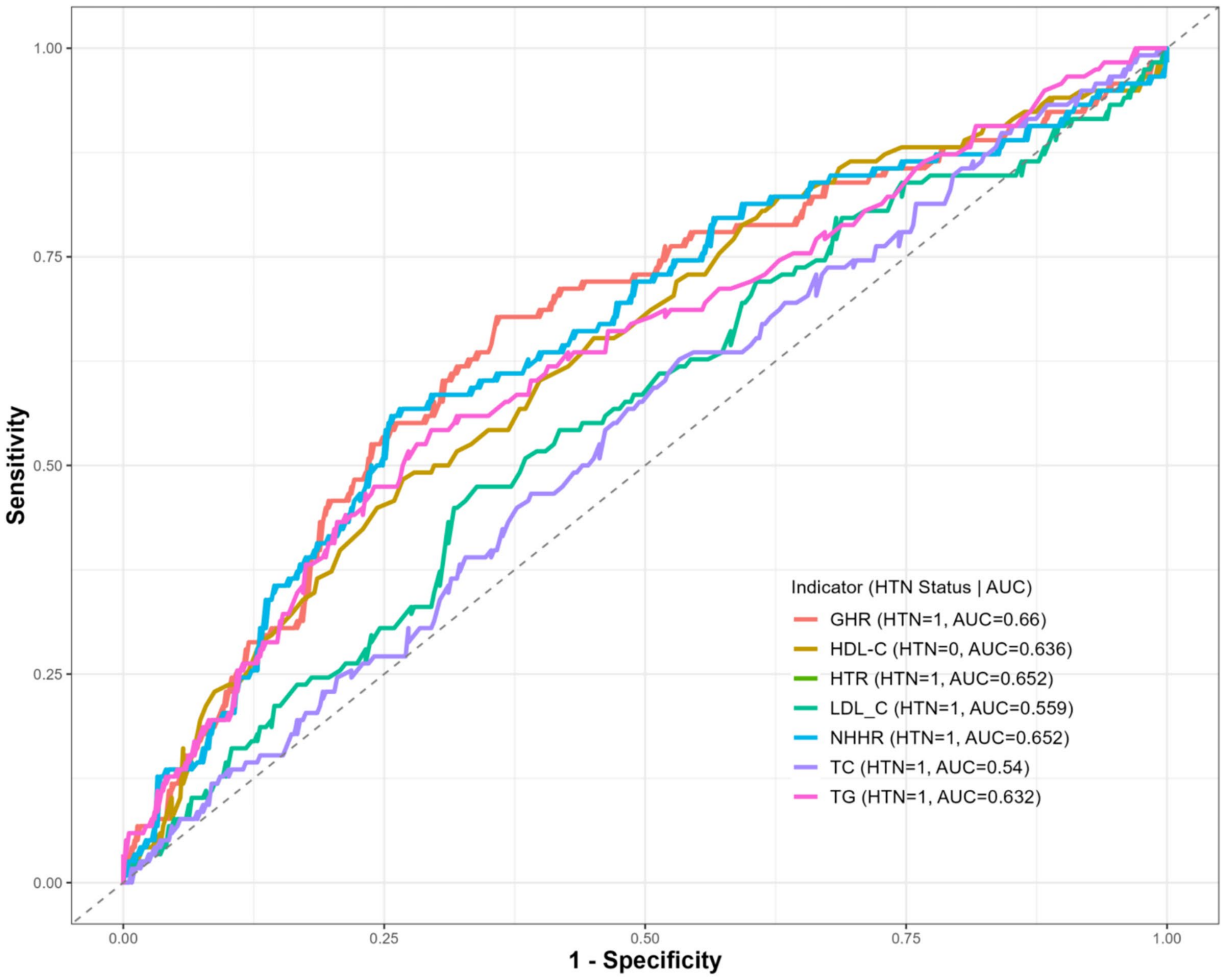
Receiver-operator characteristic curves of lipid levels and their derived indicators for predicting hypertension.

Kaplan–Meier curves (Figure 3) showed the highest cumulative hypertension risk in the high-level groups of TG, FBG/HDL-C, TC/HDL-C, and NHHR, as well as the low-level HDL-C group (all *p* < 0.001). ROC analysis (Figure 4) indicated that lipid-derived indices had better predictive performance than conventional lipids, with AUC values ranked as follows: FBG/HDL-C (0.660), TC/HDL-C (0.652), NHHR (0.652), TG (0.632), LDL-C (0.559), TC (0.540), and HDL-C (0.364). AUC differences were statistically significant (Figure 3).

RCS analysis (Supplementary Figures 3A–G) demonstrated nonlinear associations for HDL-C, FBG/HDL-C, TC/HDL-C, and NHHR (all *p* < 0.05), but not for TC, TG, or LDL-C. Segmented regression identified threshold values of 1.442 for HDL-C, 3.734 for FBG/HDL-C, 2.980 for TC/HDL-C, and 1.980 for NHHR (Table 6).

**Table 6:** Analysis of threshold effects of HDL-C, TC/HDL-C and NHHR on hypertension.

Variable	Cox regression model Adjusted *HR*(95% *CI*)	Two-stage Cox regression model Adjusted *HR*(95% *CI*)		Likelihood ratio test
HDL-C	**0.337(0.172,0.663)**	<1.442	>1.442	**0.032**
		**0.164(0.067,0.402)**	1.402(0.373,5.272)	
*p*	**0.002**	**<0.001**	0.617	
FBG/HDL-C	**1.077(1.029,1.127)**	<3.734	>3.734	0.359
		1.372(0.804,2.341)	**1.072(1.021,1.125)**	
*p*	**0.001**	0.245	0.005	
TC/HDL-C	**1.100(1.032,1.173)**	<2.980	>2.980	0.227
		0.649(0.284,1.483)	**1.109(1.042,1.181)**	
*p*	**0.004**	0.306	**0.001**	
NHHR	**1.100(1.032,1.173)**	<1.980	>1.980	0.227
		0.649(0.284,1.483)	**1.109(1.042,1.181)**	
*p*	**0.004**	0.306	**0.001**	

BMI, body mass index; TC, total cholesterol; TG, triglyceride; HDL-C, high-density lipoprotein cholesterol; LDL-C, low-density lipoprotein cholesterol; FBG/HDL-C, fasting blood glucose to high-density lipoprotein cholesterol ratio; TC/HDL-C, total cholesterol to high-density lipoprotein cholesterol ratio; NHHR, non-high-density lipoprotein cholesterol to high-density lipoprotein cholesterol ratio. Bold values indicate statistically significant results with a two-tailed *p*-value < 0.05.

#### 3.2.3 Stratified analysis

Stratified analyses revealed that the associations between lipid profiles and hypertension differed by gender, overweight status, working years, and job category.

In gender-stratified analysis (Supplementary Table 3), HDL-C, FBG/HDL-C, TC/HDL-C, and NHHR were significantly associated with hypertension in males but not females, with no significant interaction (all *p* > 0.05).

In overweight-stratified analysis (Supplementary Table 4), TG and LDL-C were significantly associated with hypertension in non-overweight individuals, while HDL-C, FBG/HDL-C, TC/HDL-C, and NHHR were significant in overweight participants. Significant interactions with overweight status were observed for LDL-C, FBG/HDL-C, TC/HDL-C, and NHHR (all *p* < 0.05).

In working-year-stratified analysis (Supplementary Table 5), HDL-C was inversely associated with hypertension in both <4.51 and ≥4.51-year groups (both *p* < 0.05), with no significant interaction. FBG/HDL-C was significant in both subgroups and showed a significant interaction with working years (*p* = 0.009). TC/HDL-C and NHHR were associated with hypertension only in the <4.51-year group (both *p* < 0.05).

In job-stratified analysis (Supplementary Table 6), TG and FBG/HDL-C were significantly associated with hypertension in industrial radiation workers, with a significant interaction between FBG/HDL-C and job type (*p* = 0.026). In medical radiation workers, HDL-C, TC/HDL-C, and NHHR were significantly associated with hypertension (all *p* < 0.05), with no significant interactions.

### 3.3 Sensitivity analysis

After excluding participants with follow-up <6 months, sensitivity analysis showed that the direction and magnitude of the associations between continuous and tertile lipid indices and hypertension risk remained largely consistent with the main findings, with the exception of triglycerides (TG), thus supporting the robustness of the results for the remaining lipid indices (Supplementary Table 7).

## 4 Discussion

This study employed a combined cross-sectional and retrospective cohort design to systematically investigate the associations among radiation exposure, dyslipidemia, and hypertension in radiation workers, and to explore potential underlying mechanisms. The findings provide empirical evidence for cardiovascular health management in populations with occupational radiation exposure.

### 4.1 Radiation exposure and hypertension risk

Cross-sectional analysis demonstrated that radiation working years and industrial radiation exposure were significantly and positively associated with prevalent hypertension, which is consistent with most previous studies conducted in occupational radiation-exposed populations (15, 35). The stronger association observed for industrial radiation (include industrial flaw detection, industrial flaw detection and well logging, etc.) relative to medical radiation may be explained by higher exposure levels, for instance, in 2022, the average annual effective dose for industrial radiography workers in Xinjiang was 0.26 mSv (36), while the average annual effective dose for nuclear medicine personnel in Beijing during the same period was only 0.185 mSv (37). Meanwhile, industrial radiation also poses an internal exposure risk (16). Existing studies have shown that the incidence of lung cancer among industrial radiation workers is higher than that among medical staff, especially for those workers who may inhale radioactive particles (38). Animal experiments have confirmed that radiation exposure impairs vasodilation in a dose-dependent manner, which may represent a key physiological mechanism underlying radiation-related hypertension (39). Only in Model 1 was it found that there was a statistically significant association between the risk of hypertension during employment and that before entry into the position. The health hazards before entry might have been influenced by lifestyle factors such as diet and exercise. As a control group, it could distinguish the health effects caused by radiation exposure from those resulting from lifestyle.

Subgroup analysis further indicated that the association between radiation exposure and hypertension was more prominent among males, individuals aged ≥35 years, drinkers, and overweight workers. The gender disparity may be attributed to higher radiation exposure opportunities and greater cumulative doses in males (40). The age-related increase in risk is linked to degradation of vascular elastic fibers and collagen deposition, which reduce vascular compliance and dilatory capacity (41, 42). Unhealthy lifestyles such as overweight and alcohol consumption may act synergistically with radiation exposure by damaging the vascular endothelium and increasing peripheral resistance (43, 44).

### 4.2 Radiation exposure and dyslipidemia

The present study confirmed a significant association between radiation exposure and dyslipidemia. Radiation exposure was positively correlated with TG levels and inversely correlated with HDL-C levels. Chronic radiation exposure contributes to elevated TC, TG, and LDL-C (45, 46), which is supported by numerous clinical and experimental studies. A cohort study in Guangdong reported that workers with ≥10 years of radiation service exhibited significantly higher TC, TG, and LDL-C than those with shorter employment (22). And the results of the non-restrictive cubic spline analysis revealed that when the working years were around 10 years, the levels of HDL-C and TC/HDL-C were the lowest, while the levels of all other lipids and their derived indicators reached a peak. This suggests that the working years in the radiology field should be controlled within 10 years. If one works in this field for a long time, the focus should be on strengthening protection and controlling lipid levels within the first 10 years after joining the company. Potential mechanisms of radiation-induced dyslipidemia may involve radiation-related adipose tissue injury and altered activity of lipid-metabolizing enzymes. Damage to perivascular adipose tissue may further disrupt blood pressure homeostasis (19, 47).

### 4.3 Mediating effect of dyslipidemia

Mediation analysis indicated that TC and LDL-C exerted partial positive mediating effects in the association between radiation working years and hypertension, whereas HDL-C played a partial negative mediating role in the relationship between job type and hypertension. Given the cross-sectional design, these findings only suggest potential biological pathways rather than definitive causal relationships (22, 48). Possible mechanisms by which dyslipidemia mediates the radiation–hypertension association include: (1) lipid deposition in the arterial endothelium leading to atherosclerosis and increased peripheral vascular resistance (14); (2) endothelial injury induced by dyslipidemia, promoting the release of vasoconstrictors and sustaining vascular contraction (32). Furthermore, animal experiments have shown that ionizing radiation can affect lipoprotein lipase (49). Some reviews have indicated that radiation causes an increase in cellular oxidative stress, damage to liver lipase activity, obstruction of fat breakdown and cholesterol recycling, accumulation of fat in internal organs, and the development of insulin resistance, leading to abnormal lipid indicators (19). This also supports the mechanism of this study.

### 4.4 Predictive value of lipid indices for hypertension

Cohort analysis showed that elevated baseline TG, fasting blood glucose (FBG)/HDL-C, TC/HDL-C, and NHHR were risk factors for incident hypertension in radiation workers, whereas higher HDL-C was protective. Non-linear associations were identified between HDL-C, FBG/HDL-C, and hypertension risk, consistent with previous reports (31, 32, 50). Sensitivity analysis revealed that only the association between TG and hypertension was unstable, which may be attributed to residual confounding, lifestyle factors, and interference from unmeasured variables.

Furthermore, this study found that HDL-C, FBG/HDL-C, TC/HDL-C and NHHR have a non-linear association with hypertension. This suggests that we should control the levels of FBG/HDL-C, TC/HDL-C and NHHR to be lower than 3.73 mmol/L, 2.98 mmol/L and 1.98 mmol/L respectively, and the HDL-C level to be higher than 1.44 mmol/L. In this way, the risk of hypertension among radiation workers will be reduced. This is consistent with the research results of Otsuka et al., which show that HDL-C has a U-shaped association with the risk of hypertension (50); it is also in line with the conclusions of the cross-sectional study of American adults, which indicates that an increase in NHHR levels can increase the risk of hypertension, and there is a non-linear association between the two (31, 32). At the same time, NHHR is also closely related to the increased risk of new-onset hypertension and heart disease in the Chinese older adults (51).

Subgroup analyses indicated that all lipid indicators except TG were significantly associated with hypertension among males. Such gender disparities may stem from inherent sex-specific differences in lipid profiles. Previous studies have reported higher TC, LDL-C and HDL-C in females, while men tend to have elevated TG and TC/HDL-C (52, 53). Endogenous estrogen exerts protective effects to sustain favorable lipid levels in women. Besides, male participants accounted for the majority of our study sample, collectively leading to more remarkable associations observed in men.

Subgroup analyses revealed that TG and LDL-C were significantly associated with hypertension exclusively in non-overweight individuals, whereas correlations between FBG/HDL-C, TC/HDL-C, NHHR and hypertension were only observed among overweight subjects. Significant multiplicative interactions were detected between overweight status and LDL-C, FBG/HDL-C, TC/HDL-C as well as NHHR, which was consistent with findings reported by Wang et al. (54). Excessive visceral fat accumulation and insulin resistance attributable to overweight can disrupt lipid homeostasis (55); owing to diminished metabolic reserve, overweight individuals are more susceptible to radiation-induced lipid derangement under equivalent occupational radiation exposure, thereby synergistically increasing hypertension susceptibility (56).

Stratified by employment duration, stronger associations of HDL-C and FBG/HDL-C with hypertension were identified in workers with ≥4.51 years of service, while TC/HDL-C and NHHR remained independently correlated with hypertension in the subgroup with service years <4.51; statistically significant interaction was only confirmed between FBG/HDL-C and length of employment. Stratification by occupational category yielded stronger associations of TG, FBG/HDL-C, TC/HDL-C and NHHR with hypertension among industrial radiation workers, whereas HDL-C was related to hypertension merely in medical radiation practitioners. Similarly, only FBG/HDL-C presented a significant interaction with job classification. Such heterogeneous subgroup results are plausibly ascribed to divergent responses of various lipid biomarkers to exposure duration and occupational radiation characteristics (22).

Predictive performance analysis revealed that lipid-derived indices (FBG/HDL-C, TC/HDL-C, NHHR) were superior to conventional lipid parameters. This advantage arises because composite indices comprehensively reflect glucose-lipid metabolic disorders and better capture overall metabolic disruption (50, 57). Subgroup analysis showed stronger associations in males and overweight individuals, which may be related to sex-specific metabolic patterns and obesity-related metabolic dysfunction (52, 54).

### 4.5 Strengths and limitations

#### 4.5.1 Strengths

The main innovations of this study are as follows: (1) The triple associations among radiation exposure, lipid metabolism, and hypertension were examined simultaneously, filling knowledge gaps regarding exposure duration and job category. (2) The mediating effects of lipid profiles were systematically analyzed, providing novel insights into the mechanisms of radiation-related hypertension. (3) The predictive performance of conventional and derived lipid indices was compared, providing practical tools for cardiovascular risk screening in occupational populations.

#### 4.5.2 Limitations

Several limitations of this study need to be addressed. First, individual accurate radiation dose data were lacking, and working years were used as a surrogate indicator of cumulative exposure, which may not fully reflect actual personal radiation levels and cause measurement bias. Second, this study was a retrospective analysis based on historical occupational physical examination archives, although the main confounding factors have been adequately adjusted, factors such as job transfer, protective measures, daily routine, sleep quality, diet (high salt, high fat), exercise, and long-term medication were not collected, and there may still be residual confounding effects that cannot be completely eliminated. Future studies should fully consider these factors. Third, the exclusion of participants with a history of hypertension, hyperlipidemia and hyperglycemia chronic diseases may have led to healthy participant bias, possibly underestimating the actual prevalence of hypertension and dyslipidemia. Single-center enrollment also restricted the generalizability of the findings. Future large-scale studies can relax the inclusion criteria and conduct stratified analysis of the data of the entire population with previous medical history. Fourth, retrospective data failed to provide detailed information on specific job types and protective measures, resulting in insufficient exposure assessment. In addition, antihypertensive and lipid-lowering medications were not adjusted for, which might interfere with relevant biochemical indicators.

### 4.6 Practical implications and future perspectives

These results carry important implications for the health management of radiation workers: (1) Strengthen radiation protection and strictly control cumulative exposure, with enhanced monitoring for industrial radiation workers. (2) Incorporate lipid indices (especially derived ratios) into routine occupational examinations to establish combined screening of lipid profiles and blood pressure. (3) Implement personalized lifestyle interventions (smoking cessation, alcohol restriction, weight management) for high-risk groups including males and overweight individuals (58, 59).

Future prospective cohort studies with objective dosimetry and multi-omics approaches are recommended to verify the observed mediating effects and clarify molecular mechanisms, supporting precise prevention and control of cardiovascular diseases in radiation workers.

## 5 Conclusion

This study explored the relationships between occupational radiation exposure, lipid profiles and hypertension in radiation workers. Increased radiation exposure was correlated with a higher hypertension risk, while different lipid markers exhibited distinct associations with hypertension. Lipid parameters may play potential indirect roles in the above relationships. Abnormal baseline lipids and lipid-derived indices could predict new-onset hypertension, with more evident effects in males and overweight individuals. These findings support the application of lipid indicators for cardiovascular risk surveillance among radiation workers. And the working years in the radiation field should be kept within 10 years; if one works for an extended period, the focus should be on strengthening protection within the first 10 years of employment, and controlling the levels of FBG/HDL-C, TC/HDL-C, and NHHR to be lower than 3.73 mmol/L, 2.98 mmol/L, and 1.98 mmol/L respectively, with the HDL-C level being higher than 1.44 mmol/L.

## Data Availability

The raw data supporting the conclusions of this article will be made available by the authors, without undue reservation.
