# Supplementary material for: Association of radiation exposure and blood lipid derived indices with hypertension risk in radiation workers
Source: Front Public Health. 2026 Jun 26;14:1870426. doi: 10.3389/fpubh.2026.1870426 (PMC13352990; doi:10.3389/fpubh.2026.1870426)
Supplement: Supplementary file 1 [file Data_Sheet_1.docx]

Supplementary Material

# Supplementary Figures and Tables

Supplementary Fig. 1 Restricted Cubic Splines of Working Years, Lipid Indices and Hypertension

Supplementary Fig. 2 Restricted Cubic Splines of Working Years and Lipid Indices

Supplementary Fig. 3 Restricted Cubic Splines of Baseline Lipid Indices and Hypertension

Supplementary Table 1 Analysis of the Association between Blood Lipids and Their Derivative Indicators and Hypertension

Supplementary Table 2 Subgroup analysis for the risk of hypertension associated with radiation

Supplementary Table 3 Gender-specific analysis of the association between blood lipids or their derived indicators and the risk of hypertension

Supplementary Table 4 Overweight stratified analysis of the association between blood lipids or their derived indicators and the risk of hypertension

Supplementary Table 5 Stratified analysis of the association between blood lipids or their derived indicators and hypertension risk by working years

Supplementary Table 6 Stratified analysis of job classification associated with blood lipids or their derived indicators and hypertension risk

Supplementary Table 7 Association analysis of continuous or the three-classification indicators of blood lipids or their derived variables with the risk of hypertension (Exclude those whose follow-up period is less than half a year)


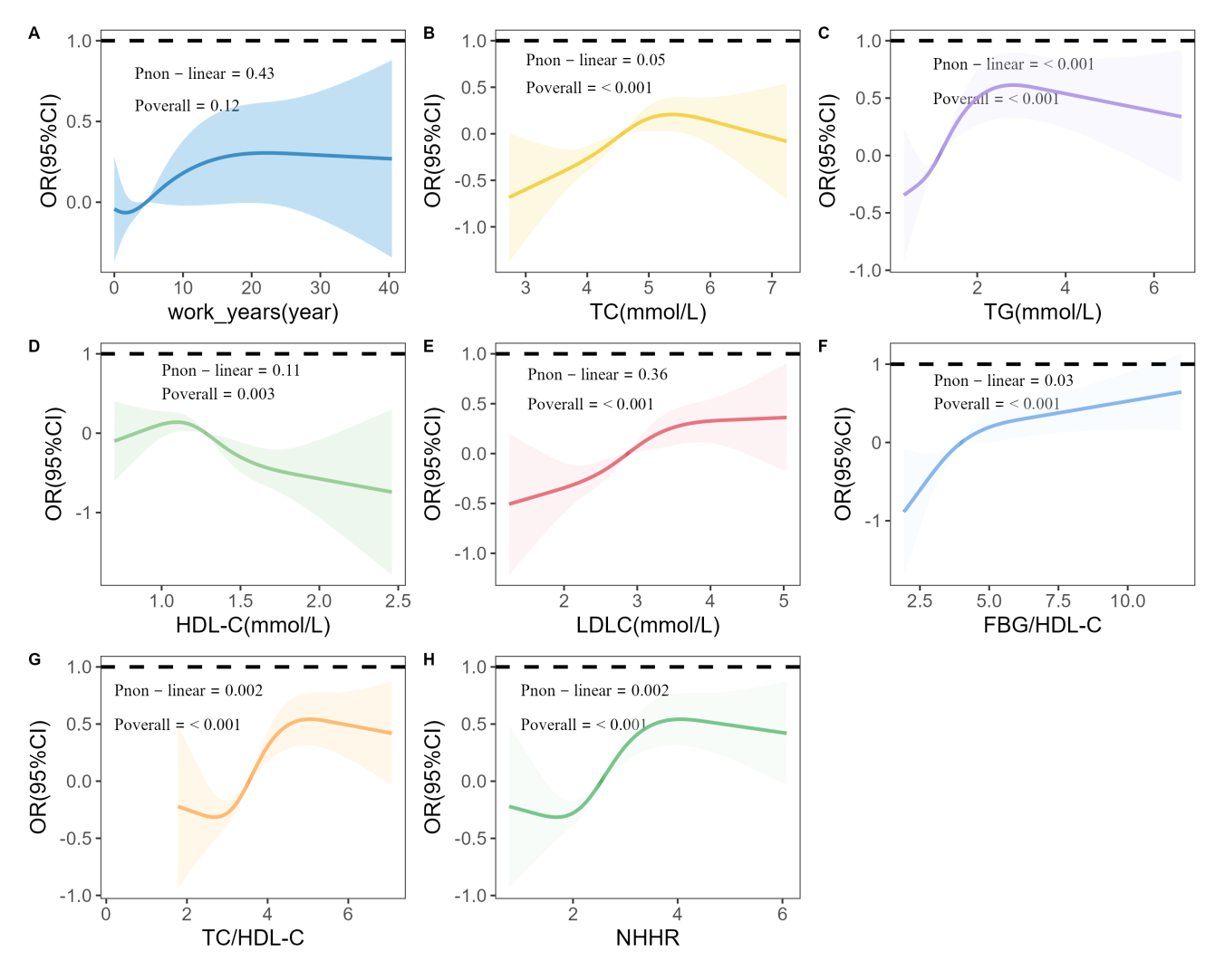


Supplementary Fig.1 Restricted Cubic Splines of Working Years, Lipid Indices and Hypertension. Restricted cubic spline analysis for the association of working years (A), TC (B), TG (C), HDL-C (D), LDL-C (E), FBG/HDL-C (F), TC/HDL-C (G), and NHHR (H) with hypertension.


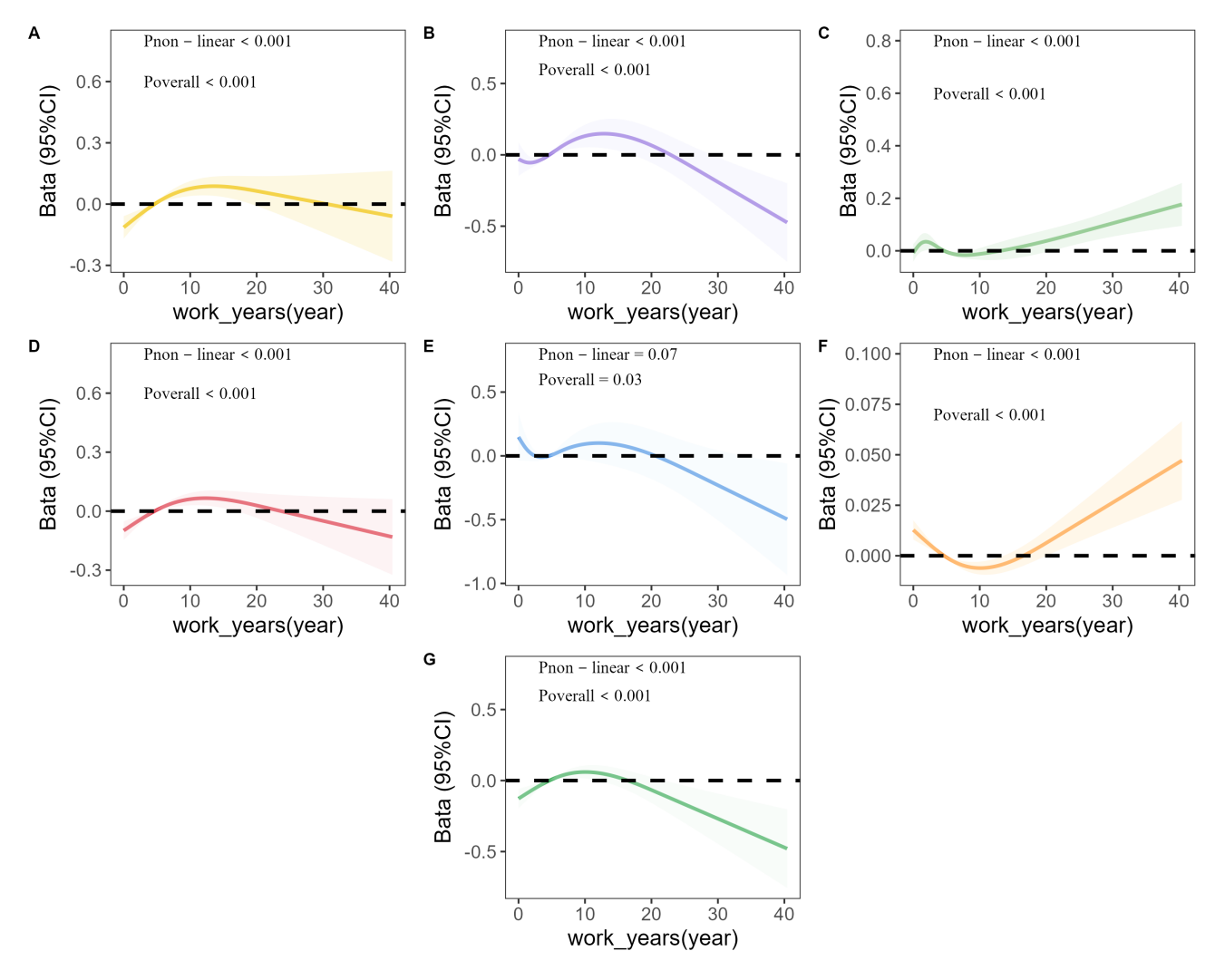


Supplementary Fig.2 Restricted Cubic Splines of Working Years and Lipid Indices. Restricted cubic spline analysis of the associations between working years and TC (A), TG (B), HDL-C (C), LDL-C (D), FBG/HDL-C (E), TC/HDL-C (F), and NHHR (G) .


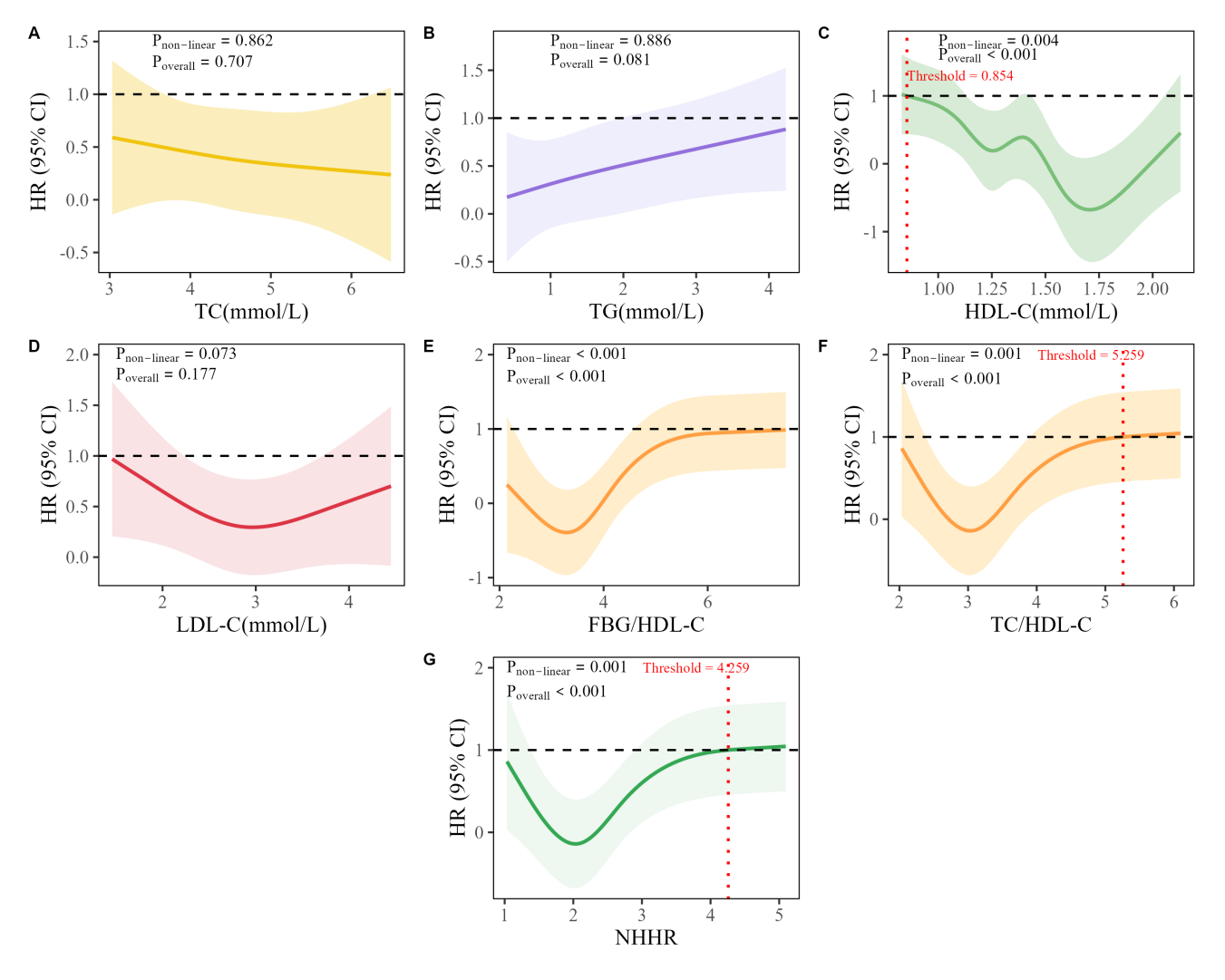


Supplementary Fig.3 Restricted Cubic Splines of Baseline Lipid Indices and Hypertension. Restricted cubic spline analysis illustrating the associations between baseline TC (A), TG (B), HDL-C (C), LDL-C (D), FBG/HDL-C (E), TC/HDL-C (F), and NHHR (G) and the risk of hypertension.

Table S1 Analysis of the Association between Blood Lipids and Their Derivative Indicators and Hypertension

| **Blood Lipids and Their Derivative Indicators** | **Hypertension *OR*(95% *CI*)** | | |
| --- | --- | --- | --- |
|  | **Model 1** | **Model 2** | **Model 3** |
| **TC** | **1.410(1.267,1.571)** | **1.270(1.130,1.427)** | **1.235(1.095,1.393)** |
| **TG** | **1.512(1.389,1.648)** | **1.229(1.124,1.344)** | **1.202(1.099,1.317)** |
| **HDL-C** | **0.208(0.148,0.291)** | **0.485(0.333,0.700)** | **0.538(0.366,0.787)** |
| **LDL-C** | **1.725(1.523,1.956)** | **1.422(1.242,1.628)** | **1.361(1.183,1.566)** |
| **FBG/HDL-C** | **1.367(1.280,1.463)** | **1.147(1.079,1.226)** | **1.128(1.062,1.206)** |
| **TC/HDL-C** | **1.650(1.507,1.808)** | **1.287(1.168,1.420)** | **1.229(1.115,1.360)** |
| **NHHR** | **1.650(1.507,1.808)** | **1.287(1.167,1.420)** | **1.229(1.115,1.360)** |
| **TC** **groups** |  |  |  |
| Low (<4.21) | Reference | Reference | Reference |
| Middle (4.21-4.95) | **1.546(1.209,1.981)** | **1.401(1.081,1.818)** | **1.344(1.031,1.756)** |
| High (>4.95) | **2.288(1.808,2.906)** | **1.724(1.340,2.224)** | **1.631(1.259,2.118)** |
| **TG** **groups** |  |  |  |
| Low (<0.87) | Reference | Reference | Reference |
| Middle (0.87-1.52) | **2.147(1.643,2.821)** | **1.620(1.224,2.155)** | **1.539(1.157,2.057)** |
| High (>1.52) | **4.282(3.328,5.553)** | **2.464(1.882,3.246)** | **2.230(1.693,2.955)** |
| **HDL-C groups** |  |  |  |
| Low (<1.16) | Reference | Reference | Reference |
| Middle (1.16-1.43) | **0.787(0.636,0.974)** | 0.955(0.764,1.195) | 1.001(0.795,1.261) |
| High (>1.43) | **0.324(0.250,0.418)** | **0.593(0.447,0.783)** | **0.622(0.464,0.830)** |
| **LDL-C groups** |  |  |  |
| Low (<2.57) | Reference | Reference | Reference |
| Middle (2.57-3.21) | **1.857(1.442,2.401)** | **1.447(1.108,1.895)** | **1.395(1.063,1.834)** |
| High (>3.21) | **2.973(2.332,3.810)** | **1.992(1.538,2.590)** | **1.819(1.395,2.379)** |
| **FBG/HDL-C groups** |  |  |  |
| Low (<3.53) | Reference | Reference | Reference |
| Middle (3.53-4.52) | **2.346(1.802,3.071)** | **1.565(1.183,2.079)** | **1.523(1.143,2.038)** |
| High (>4.52) | **3.899(3.028,5.057)** | **1.942(1.474,2.572)** | **1.826(1.375,2.439)** |
| **TC/HDL-C groups** |  |  |  |
| Low (<3.12) | Reference | Reference | Reference |
| Middle (3.12-3.99) | **2.124(1.624,2.792)** | **1.438(1.083,1.919)** | **1.356(1.015,1.819)** |
| High (>3.99) | **4.327(3.359,5.617)** | **2.221(1.687,2.940)** | **2.007(1.515,2.672)** |
| **NHHR groups** |  |  |  |
| Low (<2.12) | Reference | Reference | Reference |
| Middle (2.12-2.99) | **2.124(1.624,2.792)** | **1.438(1.083,1.919)** | **1.356(1.015,1.819)** |
| High (>2.99) | **4.327(3.359,5.617)** | **2.221(1.687,2.940)** | **2.007(1.515,2.672)** |

Model 1: Unadjusted model; Model 2: Adjusted for sex and age; Model 3: Further adjusted for BMI, education level, smoking status, and alcohol consumption on the basis of Model 2.

Table S2 Subgroup analysis of radiation exposure and the risk of hypertension

| **Subgroup variables** | **Working years** | ***P*-interaction** | **Working years groups** | | ***P*-interaction** | **Type of physical examination** | | ***P*-interaction** | **Job classification** | ***P*-interaction** |
| --- | --- | --- | --- | --- | --- | --- | --- | --- | --- | --- |
|  |  |  | Middle **^a^** | High **^a^** |  | During employment **^b^** | Post-employment **^b^** |  | Industrial **^c^** |  |
| **Sex** |  | 0.609 |  |  | 0.222 |  |  | 0.474 |  | 0.751 |
| Female | 0.981  (0.950,1.012) |  | 1.349  (0.752,2.442) | 0.871  (0.463,1.638) |  | 0.876  (0.511,1.546) | **0.234**  **(0.035,0.886)** |  | **2.300**  **(1.265,4.082)** |  |
| Male | **1.020**  **(1.006,1.034)** |  | 1.007  (0.753,1.346) | **1.599**  **(1.191,2.152)** |  | 1.268  (0.945,1.714) | 0.863  (0.463,1.558) |  | **1.869**  **(1.471,2.381)** |  |
| **Age groups** |  | 0.423 |  |  | 0.846 |  |  | 0.261 |  | 0.985 |
| <35 | 1.031  (0.986,1.077) |  | 1.016  (0.713,1.449) | 1.409  (0.834,2.338) |  | 1.247  (0.857,1.835) | 0.415  (0.119,1.111) |  | **1.662**  **(1.175,2.368)** |  |
| ≥35 | **1.017**  **(1.005,1.030)** |  | 1.209  (0.822,1.782) | **1.566**  **(1.131,2.186)** |  | 1.210  (0.849,1.747) | 1.039  (0.529,1.980) |  | **1.854**  **(1.407,2.441)** |  |
| **Educational level** |  | 0.706 |  |  | 0.554 |  |  | 0.731 |  | 0.500 |
| Junior high school or below | 1.012  (0.992,1.031) |  | 1.040  (0.706,1.536) | 1.417  (0.944,2.135) |  | 1.207  (0.808,1.830) | 0.863  (0.395,1.800) |  | **2.170**  **(1.562,3.026)** |  |
| Senior high school / polytechnic | 1.017  (0.996,1.038) |  | 1.059  (0.692,1.623) | **1.726**  **(1.130,2.654)** |  | 1.356  (0.882,2.130) | 0.648  (0.205,1.710) |  | **1.621**  **(1.152,2.288)** |  |
| Junior college or above | 1.002  (0.973,1.031) |  | 1.208  (0.637,2.277) | 0.911  (0.470,1.748) |  | 0.828  (0.471,1.467) | 0.402  (0.079,1.509) |  | **2.242**  **(1.165,4.282)** |  |
| **Smoking** |  | 0.232 |  |  | 0.084 |  |  | 0.543 |  | 0.614 |
| No | **1.017**  **(1.003,1.032)** |  | 1.231  (0.918,1.652) | **1.603**  **(1.193,2.159)** |  | 1.238  (0.926,1.669) | 0.805  (0.432,1.440) |  | **2.005**  **(1.567,2.570)** |  |
| Yes | 0.993  (0.966,1.021) |  | 0.616  (0.341,1.101) | 0.834  (0.434,1.578) |  | 0.986  (0.546,1.807) | 0.381  (0.077,1.411) |  | **1.650**  **(1.001,2.741)** |  |
| **Alcohol drinking** |  | 0.355 |  |  | 0.229 |  |  | 0.488 |  | 0.518 |
| No | 1.000  (0.984,1.017) |  | 1.125  (0.802,1.579) | 1.147  (0.817,1.614) |  | 1.032  (0.744,1.444) | 0.537  (0.238,1.109) |  | **1.911**  **(1.430,2.557)** |  |
| Yes | **1.032**  **(1.012,1.054)** |  | 1.042  (0.692,1.574) | **2.097**  **(1.360,3.260)** |  | 1.486  (0.972,2.316) | 1.037  (0.445,2.300) |  | **1.958**  **(1.388,2.778)** |  |
| **Overweight** |  | 0.776 |  |  | 0.331 |  |  | 0.005 |  | 0.279 |
| No | 1.013  (0.989,1.038) |  | 0.833  (0.512,1.351) | 1.388  (0.867,2.237) |  | 0.663  (0.431,1.028) | 0.361  (0.114,0.937) |  | **2.479**  **(1.652,3.734)** |  |
| Yes | 1.011  (0.996,1.026) |  | 1.182  (0.868,1.611) | **1.418**  **(1.031,1.954)** |  | **1.663**  **(1.201,2.332)** | 0.971  (0.490,1.848) |  | **1.731**  **(1.333,2.253)** |  |

^a^ For working years groups, the medium and high level groups were both referenced to the low level group;  ^b^ For type of physical examination, during employment and post-employment were both referenced to pre-employment;  ^c^ For job classification, industrial radiation workers were referenced to medical radiation workers.

Table S3 Gender-specific analysis of the association between blood lipids or their derived indicators and the risk of hypertension

| **Blood Lipids and Their Derivative Indicators** | **Hypertension**  ***n*(%)** | ***HR*(95% *CI*)** | | |
| --- | --- | --- | --- | --- |
|  |  | **Model 1** | **Model 2** | **Model 3** |
| **TC** |  |  |  |  |
| Female | 27(12.8) | 1.269(0.813,1.980) | 0.822(0.500,1.353) | 0.720(0.426,1.216) |
| Male | 91(33.3) | 0.978(0.757,1.264) | 0.867(0.654,1.151) | 0.924(0.698,1.224) |
| *P*-interaction |  | 0.393 | 0.329 | 0.354 |
| **TG** |  |  |  |  |
| Female | 27(12.8) | **1.683(1.047,2.704)** | 1.223(0.705,2.122) | 1.228(0.690,2.188) |
| Male | 91(33.3) | **1.221(1.047,1.423)** | **1.185(1.011,1.388)** | 1.165(0.985,1.377) |
| *P*-interaction |  | 0.235 | 0.325 | 0.315 |
| **HDL-C** |  |  |  |  |
| Female | 27(12.8) | 0.793(0.241,2.613) | 1.054(0.311,3.572) | 0.726(0.200,2.629) |
| Male | 91(33.3) | **0.285(0.136,0.597)** | **0.268(0.126,0.570)** | **0.255(0.113,0.574)** |
| *P*-interaction |  | 0.211 | 0.142 | 0.114 |
| **LDL-C** |  |  |  |  |
| Female | 27(12.8) | 1.203(0.715,2.026) | 0.689(0.382,1.243) | 0.630(0.338,1.172) |
| Male | 91(33.3) | 1.031(0.746,1.424) | 0.908(0.645,1.279) | 0.992(0.704,1.397) |
| *P*-interaction |  | 0.681 | 0.616 | 0.706 |
| **FBG/HDL-C** |  |  |  |  |
| Female | 27(12.8) | 1.293(0.905,1.848) | 1.084(0.699,1.682) | 1.247(0.806,1.931) |
| Male | 91(33.3) | **1.058(1.016,1.101)** | **1.062(1.018,1.108)** | **1.082(1.032,1.135)** |
| *P*-interaction |  | 0.231 | 0.359 | 0.387 |
| **TC/HDL-C** |  |  |  |  |
| Female | 27(12.8) | 1.458(0.958,2.220) | 1.050(0.627,1.758) | 1.134(0.656,1.962) |
| Male | 91(33.3) | **1.068(1.007,1.133)** | **1.075(1.009,1.144)** | **1.107(1.034,1.185)** |
| *P*-interaction |  | 0.157 | 0.336 | 0.361 |
| **NHHR** |  |  |  |  |
| Female | 27(12.8) | 1.458(0.958,2.220) | 1.05(0.627,1.758) | 1.126(0.650,1.950) |
| Male | 91(33.3) | **1.068(1.007,1.133)** | **1.075(1.009,1.144)** | **1.107(1.034,1.185)** |
| *P*-interaction |  | 0.157 | 0.336 | 0.367 |

Model 1: Unadjusted; Model 2: Adjusted for age; Model 3: Further adjusted for BMI, educational level, smoking, alcohol drinking, and job category on the basis of Model 2.

Table S4 Overweight stratified analysis of the association between blood lipids or their derived indicators and the risk of hypertension

| **Blood Lipids and Their Derivative Indicators** | **Hypertension**  ***n*(%)** | ***HR*(95% *CI*)** | | |
| --- | --- | --- | --- | --- |
|  |  | **Model 1** | **Model 2** | **Model 3** |
| **TC** |  |  |  |  |
| No | 40(20.0) | 0.894(0.604,1.323) | 0.665(0.429,1.030) | 0.635(0.395,1.021) |
| Yes | 78(27.5) | 1.132(0.877,1.461) | 1.008(0.760,1.338) | 1.083(0.809,1.450) |
| *P*-interaction |  | 0.302 | 0.172 | 0.140 |
| **TG** |  |  |  |  |
| No | 40(20.0) | **1.492(1.105,2.015)** | 1.388(0.994,1.938) | **1.449(1.018,2.063)** |
| Yes | 78(27.5) | **1.277(1.093,1.492)** | 1.122(0.945,1.334) | 1.150(0.960,1.379) |
| *P*-interaction |  | 0.395 | 0.571 | 0.639 |
| **HDL-C** |  |  |  |  |
| No | 40(20.0) | 0.422(0.170,1.047) | 0.384(0.130,1.129) | 0.363(0.126,1.044) |
| Yes | 78(27.5) | **0.201(0.088,0.460)** | **0.338(0.144,0.792)** | **0.288(0.120,0.690)** |
| *P*-interaction |  | 0.266 | 0.261 | 0.206 |
| **LDL-C** |  |  |  |  |
| No | 40(20.0) | 0.904(0.582,1.404) | 0.613(0.369,1.018) | **0.562(0.319,0.989)** |
| Yes | 78(27.5) | 1.350(0.971,1.878) | 1.113(0.779,1.590) | 1.230(0.853,1.775) |
| *P*-interaction |  | 0.143 | **0.045** | **0.021** |
| **FBG/HDL-C** |  |  |  |  |
| No | 40(20.0) | **1.054(1.000,1.110)** | **1.067(1.006,1.131)** | 1.055(0.992,1.121) |
| Yes | 78(27.5) | **1.378(1.226,1.548)** | **1.247(1.082,1.436)** | **1.276(1.110,1.467)** |
| *P*-interaction |  | **<0.001** | **0.034** | **0.022** |
| **TC/HDL-C** |  |  |  |  |
| No | 40(20.0) | 1.070(0.995,1.150) | 1.082(0.996,1.176) | 1.064(0.976,1.159) |
| Yes | 78(27.5) | **1.413(1.201,1.663)** | **1.262(1.046,1.524)** | **1.426(1.157,1.757)** |
| *P*-interaction |  | **0.004** | **0.045** | **0.011** |
| **NHHR** |  |  |  |  |
| No | 40(20.0) | 1.070(0.995,1.150) | 1.082(0.996,1.176) | 1.064(0.976,1.159) |
| Yes | 78(27.5) | **1.413(1.201,1.663)** | **1.262(1.046,1.524)** | **1.426(1.157,1.756)** |
| *P*-interaction |  | **0.004** | **0.045** | **0.011** |

Model 1: Unadjusted; Model 2: Adjusted for sex and age; Model 3: Further adjusted for educational level, smoking, alcohol drinking and job category based on Model 2.

Table S5 Stratified analysis of the association between blood lipids or their derived indicators and hypertension risk by working years

| **Blood Lipids and Their Derivative Indicators** | **Hypertension**  ***n*(%)** | ***HR*(95% *CI*)** | | |
| --- | --- | --- | --- | --- |
|  |  | **Model 1** | **Model 2** | **Model 3** |
| **TC** |  |  |  |  |
| <4.51 years | 52(21.5) | 1.213(0.848,1.735) | 1.135(0.775,1.661) | 1.126(0.763,1.663) |
| ≥4.51 years | 66(27.3) | 0.876(0.644,1.192) | 0.716(0.511,1.005) | 0.722(0.512,1.017) |
| *P*-interaction |  | 0.189 | 0.141 | 0.156 |
| **TG** |  |  |  |  |
| <4.51 years | 52(21.5) | **1.555(1.174,2.061)** | 1.349(0.975,1.868) | 1.317(0.942,1.840) |
| ≥4.51 years | 66(27.3) | **1.269(1.076,1.497)** | 1.159(0.970,1.385) | 1.161(0.963,1.400) |
| *P*-interaction |  | 0.224 | 0.672 | 0.685 |
| **HDL-C** |  |  |  |  |
| <4.51 years | 52(21.5) | **0.284(0.123,0.652)** | 0.381(0.144,1.009) | **0.309(0.110,0.869)** |
| ≥4.51 years | 66(27.3) | **0.250(0.108,0.581)** | **0.297(0.119,0.742)** | **0.286(0.110,0.742)** |
| *P*-interaction |  | 0.914 | 0.643 | 0.811 |
| **LDL-C** |  |  |  |  |
| <4.51 years | 52(21.5) | 1.399(0.943,2.077) | 1.224(0.797,1.881) | 1.243(0.799,1.933) |
| ≥4.51 years | 66(27.3) | 0.931(0.646,1.343) | 0.696(0.468,1.034) | 0.701(0.465,1.054) |
| *P*-interaction |  | 0.143 | 0.119 | 0.126 |
| **FBG/HDL-C** |  |  |  |  |
| <4.51 years | 52(21.5) | **1.059(1.009,1.111)** | 1.049(0.993,1.108) | **1.066(1.006,1.131)** |
| ≥4.51 years | 66(27.3) | **1.619(1.375,1.906)** | **1.404(1.151,1.712)** | **1.463(1.182,1.810)** |
| *P*-interaction |  | **<0.001** | **0.005** | **0.009** |
| **TC/HDL-C** |  |  |  |  |
| <4.51 years | 52(21.5) | **1.084(1.019,1.152)** | **1.072(1.000,1.148)** | **1.096(1.016,1.181)** |
| ≥4.51 years | 66(27.3) | **1.354(1.110,1.651)** | 1.241(0.976,1.578) | 1.255(0.976,1.614) |
| *P*-interaction |  | 0.051 | 0.252 | 0.268 |
| **NHHR** |  |  |  |  |
| <4.51 years | 52(21.5) | **1.084(1.019,1.152)** | **1.072(1.000,1.148)** | **1.096(1.016,1.181)** |
| ≥4.51 years | 66(27.3) | **1.354(1.110,1.651)** | 1.241(0.976,1.578) | 1.255(0.976,1.6131) |
| *P*-interaction |  | 0.051 | 0.252 | 0.272 |

Model 1: Unadjusted; Model 2: Adjusted for sex and age; Model 3: Further adjusted for BMI, educational level, smoking, alcohol drinking and job category on the basis of Model 2.

Table S6 Stratified analysis of job classification associated with blood lipids or their derived indicators and hypertension risk

| **Blood Lipids and Their Derivative Indicators** | **Hypertension**  ***n*(%)** | ***HR*(95% *CI*)** | | | | |
| --- | --- | --- | --- | --- | --- | --- |
|  |  | **Model 1** | **Model 2** | | **Model 3** | |
| **TC** |  |  |  |  | |  |
| Medical | 66(22.8) | 1.144(0.865,1.513) | 0.909(0.660,1.251) | 0.919(0.664,1.271) | |  |
| Industrial | 52(26.7) | 0.931(0.656,1.320) | 0.865(0.591,1.265) | 0.909(0.624,1.323) | |  |
| *P*-interaction |  | 0.382 | 0.428 | 0.473 | |  |
| **TG** |  |  |  |  | |  |
| Medical | 66(22.8) | **1.479(1.209,1.811)** | 1.156(0.910,1.468) | 1.143(0.892,1.465) | |  |
| Industrial | 52(26.7) | **1.261(1.041,1.528)** | 1.219(0.994,1.493) | **1.264(1.017,1.569)** | |  |
| *P*-interaction |  | 0.271 | 0.818 | 0.949 | |  |
| **HDL-C** |  |  |  |  | |  |
| Medical | 66(22.8) | **0.213(0.102,0.442)** | **0.303(0.129,0.712)** | **0.298(0.124,0.719)** | |  |
| Industrial | 52(26.7) | **0.371(0.144,0.952)** | 0.411(0.145,1.167) | 0.374(0.133,1.054) | |  |
| *P*-interaction |  | 0.401 | 0.474 | 0.418 | |  |
| **LDL-C** |  |  |  |  | |  |
| Medical | 66(22.8) | 1.335(0.948,1.880) | 0.960(0.663,1.390) | 0.983(0.671,1.441) | |  |
| Industrial | 52(26.7) | 0.970(0.642,1.465) | 0.854(0.542,1.346) | 0.916(0.578,1.451) | |  |
| *P*-interaction |  | 0.257 | 0.303 | 0.316 | |  |
| **FBG/HDL-C** |  |  |  |  | |  |
| Medical | 66(22.8) | **1.067(1.023,1.112)**) | 1.054(0.996,1.115) | 1.058(0.996,1.123) | |  |
| Industrial | 52(26.7) | **1.338(1.158,1.547)** | **1.321(1.131,1.544)** | **1.332(1.137,1.561)** | |  |
| *P*-interaction |  | **0.006** | **0.023** | **0.026** | |  |
| **TC/HDL-C** |  |  |  |  | |  |
| Medical | 66(22.8) | **1.090(1.031,1.152)** | **1.084(1.010,1.164)** | **1.097(1.018,1.182)** | |  |
| Industrial | 52(26.7) | **1.311(1.039,1.653)** | 1.257(0.973,1.624) | **1.382(1.048,1.822)** | |  |
| *P*-interaction |  | 0.126 | 0.488 | 0.432 | |  |
| **NHHR** |  |  |  |  | |  |
| Medical | 66(22.8) | **1.090(1.031,1.152)** | **1.084(1.010,1.164)** | **1.097(1.018,1.182)** | |  |
| Industrial | 52(26.7) | **1.311(1.039,1.653)** | 1.257(0.973,1.624) | **1.380(1.047,1.820)** | |  |
| *P*-interaction |  | 0.126 | 0.488 | 0.439 | |  |

Model 1: Unadjusted; Model 2: Adjusted for sex and age; Model 3: Further adjusted for educational level, smoking and alcohol drinking on the basis of Model 2.

Table S7 Association analysis of continuous or the three-classification indicators of blood lipids or their derived variables with the risk of hypertension (Exclude those whose follow-up period is less than half a year)

| **Blood Lipids and Their Derivative Indicators(mmol/L)** | **Number of participants** | **Cases / person-years** | ***HR*(95% *CI*)** | | |
| --- | --- | --- | --- | --- | --- |
|  |  |  | **Model 1** | **Model 2** | **Model 3** |
| **TC** | 440 | 108/670.4 | 1.042(0.826,1.313) | 0.910(0.704,1.177) | 0.936(0.722,1.215) |
| **TG** | 440 | 108/670.4 | **1.316(1.138,1.521)** | 1.163(0.988,1.368) | 1.157(0.978,1.367) |
| **HDL-C** | 440 | 108/670.4 | **0.280(0.152,0.514)** | **0.410(0.208,0.811)** | **0.393(0.194,0.793)** |
| **LDL-C** | 440 | 108/670.4 | 1.171(0.887,1.545) | 0.927(0.681,1.260) | 0.970(0.710,1.327) |
| **FBG/HDL-C** | 440 | 108/670.4 | **1.075(1.036,1.115)** | **1.063(1.018,1.110)** | **1.072(1.024,1.123)** |
| **TC/HDL-C** | 440 | 108/670.4 | **1.095(1.039,1.153)** | **1.076(1.010,1.146)** | **1.090(1.020,1.164)** |
| **NHHR** | 440 | 108/670.4 | **1.095(1.039,1.153)** | **1.076(1.008,1.146)** | **1.090(1.020,1.164)** |
| **TC groups** |  |  |  |  |  |
| Low (<4.21) | 141 | 31/215 | Reference | Reference | Reference |
| Middle (4.21-4.91) | 151 | 37/226 | 1.196(0.741,1.928) | 1.028(0.635,1.666) | 1.010(0.618,1.651) |
| High (>4.91) | 148 | 40/229 | 1.122(0.700,1.799) | 0.838(0.506,1.389) | 0.864(0.519,1.438) |
| *P* for trend |  |  | 0.872 | 0.980 | 0.928 |
| **TG groups** |  |  |  |  |  |
| Low (<0.83) | 147 | 26/222 | Reference | Reference | Reference |
| Middle (0.83-1.39) | 146 | 26/223 | 1.007(0.585,1.735) | 0.822(0.474,1.427) | 0.819(0.471,1.422) |
| High (>1.39) | 147 | 56/225 | **2.148(1.347,3.426)** | 1.412(0.855,2.334) | 1.418(0.855,2.351) |
| *P* for trend |  |  | 0.866 | 0.772 | 0.701 |
| **HDL-C groups** |  |  |  |  |  |
| Low (<1.20) | 144 | 53/212 | Reference | Reference | Reference |
| Middle (1.20-1.50) | 141 | 32/213 | **0.566(0.364,0.878)** | **0.639(0.410,0.997)** | **0.612(0.390,0.961)** |
| High (>1.50) | 155 | 23/245 | **0.315(0.191,0.519)** | **0.419(0.246,0.712)** | **0.412(0.241,0.705)** |
| *P* for trend |  |  | 0.343 | 0.359 | 0.324 |
| **LDL-C groups** |  |  |  |  |  |
| Low (<2.56) | 142 | 27/213 | Reference | Reference | Reference |
| Middle (2.56-3.14) | 150 | 37/224 | 1.319(0.802,2.167) | 0.946(0.566,1.580) | 0.977(0.583,1.638) |
| High (>3.14) | 148 | 44/233 | 1.337(0.826,2.167) | 0.913(0.545,1.528) | 0.971(0.577,1.1633) |
| *P* for trend |  |  | 0.643 | 0.788 | 0.867 |
| **FBG/HDL-C groups** |  |  |  |  |  |
| Low (<3.38) | 153 | 25/235 | Reference | Reference | Reference |
| Middle (3.38-4.33) | 143 | 24/219 | 1.061(0.602,1.869) | 0.869(0.487,1.552) | 0.840(0.467,1.511) |
| High (>4.33) | 144 | 59/216 | **2.950(1.829,4.757)** | **1.976(1.164,3.352)** | **1.969(1.141,3.398)** |
| *P* for trend |  |  | 0.199 | 0.241 | 0.239 |
| **TC/HDL-C groups** |  |  |  |  |  |
| Low (<3.01) | 147 | 20/225 | Reference | Reference | Reference |
| Middle (3.01-3.75) | 149 | 28/229 | 1.341(0.755,2.381) | 1.068(0.593,1.924) | 1.065(0.589,1.925) |
| High (>3.75) | 144 | 60/217 | **3.201(1.927,5.317)** | **2.154(1.242,3.735)** | **2.223(1.280,3.859)** |
| *P* for trend |  |  | 0.302 | 0.276 | 0.236 |
| **NHHR groups** |  |  |  |  |  |
| Low (<2.01) | 147 | 20/225 | Reference | Reference | Reference |
| Middle (2.01-2.75) | 148 | 28/228 | 1.341(0.755,2.381) | 1.068(0.593,1.924) | 1.065(0.589,1.925) |
| High (>2.75) | 145 | 60/217 | **3.201(1.927,5.317)** | **2.153(1.242,3.735)** | **2.223(1.280,3.859)** |
| *P* for trend |  |  | 0.301 | 0.276 | 0.185 |

Model 1: Unadjusted; Model 2: Adjusted for sex and age; Model 3: Further adjusted for educational level, BMI, smoking, alcohol drinking and job category on the basis of Model 2.
